# Enhancing high-fidelity nonlinear solver with reduced order model

**DOI:** 10.1038/s41598-022-22407-6

**Published:** 2022-11-23

**Authors:** Teeratorn Kadeethum, Daniel O’Malley, Francesco Ballarin, Ida Ang, Jan N. Fuhg, Nikolaos Bouklas, Vinicius L. S. Silva, Pablo Salinas, Claire E. Heaney, Christopher C. Pain, Sanghyun Lee, Hari S. Viswanathan, Hongkyu Yoon

**Affiliations:** 1 Sandia National Laboratories, Albuquerque, NM 87185 USA; 2 Los Alamos National Laboratory, Los Alamos, NM 87545 USA; 3 Università Cattolica del Sacro Cuore, 25133 Brescia, Italy; 4 Cornell University, Ithaca, NY 14853 USA; 5 Imperial College London, London, SW7 2AZ UK; 6 Florida State University, Tallahassee, FL 32306 USA

**Keywords:** Soft materials, Hydrology, Solid Earth sciences

## Abstract

We propose the use of reduced order modeling (ROM) to reduce the computational cost and improve the convergence rate of nonlinear solvers of full order models (FOM) for solving partial differential equations. In this study, a novel ROM-assisted approach is developed to improve the computational efficiency of FOM nonlinear solvers by using ROM’s prediction as an initial guess. We hypothesize that the nonlinear solver will take fewer steps to the converged solutions with an initial guess that is closer to the real solutions. To evaluate our approach, four physical problems with varying degrees of nonlinearity in flow and mechanics have been tested: Richards’ equation of water flow in heterogeneous porous media, a contact problem in a hyperelastic material, two-phase flow in layered porous media, and fracture propagation in a homogeneous material. Overall, our approach maintains the FOM’s accuracy while speeding up nonlinear solver by 18–73% (through suitable ROM-assisted FOMs). More importantly, the proximity of ROM’s prediction to the solution space leads to the improved convergence of FOMs that would have otherwise diverged with default initial guesses. We demonstrate that the ROM’s accuracy can impact the computational efficiency with more accurate ROM solutions, resulting in a better cost reduction. We also illustrate that this approach could be used in many FOM discretizations (e.g., finite volume, finite element, or a combination of those). Since our ROMs are data-driven and non-intrusive, the proposed procedure can easily lend itself to any nonlinear physics-based problem.

## Introduction

Many natural and engineering processes ranging from subsurface flow and mechanical physics, aerospace engineering to material science are governed by partial differential equations (PDEs)^1–6^. The PDEs can be solved analytically for simple geometries and boundary conditions. For complex problems with non-homogeneous boundary and initial conditions, various geometries, and/or material properties, however, numerical approximations such as finite difference, finite volume, or finite element methods, referred to as full order model (FOM) hereafter, are primarily used to solve these governing equations^7^. Although the FOMs have been widely used, they require substantial computational resources, making them not practically suitable for handling large-scale inverse problems, optimization, or control, in which an extensive set of simulations must be explored^8–10^. Besides, as PDEs become nonlinear, the solver used to approximate usually takes considerable time to converge or, in the worst case, does not converge at all.

Reduced order modeling (ROM) is emerging as an alternative that provides a reasonable accuracy while requiring a much lower computational cost compared to the FOM^9,11^. In this work, ROM is suitable for a parameterized problem, where the problem is repeatedly evaluated with a set of parameters $\varvec{\mu }$ such as physical properties, geometric characteristics, or boundary conditions^9,12^. ROM is generally composed of two stages, the offline and online stages. The offline stage begins with the initialization of the set of $\varvec{\mu }$. The FOM is then solved for each member of $\varvec{\mu }$. Dimensional reduction techniques are used to compress the data from the previous step to produce linear or nonlinear reduced manifolds^13^ that span a reduced space of very low dimensionality but with enough accuracy for a reproduction of the FOM^14,15^. During the online or prediction phase, ROM can deliver an approximation of FOM for any desired value of $\varvec{\mu }$ by seeking a latent representation in the reduced manifolds and then reconstructing this proxy to the high-fidelity solution space.

There are generally two types of ROM; intrusive and non-intrusive ROM. An intrusive ROM often relies on proper orthogonal decomposition (POD) as a linear compression tool. However, nonlinear manifolds have recently been incorporated into intrusive ROMs (with specialized linearizations) for PDEs^16^ and generalized eigenvalue problems^17^. The reconstruction of FOM from linear reduced manifolds is completed through either Galerkin or Petrov-Galerkin projection^9,18,19^. This approach has the primary advantage of preserving physical laws and requiring less training data. The non-intrusive (data-driven) ROM, on the other hand, can straightforwardly utilize both linear and nonlinear reduced manifolds constructed by POD or an autoencoder^13,20–26^ interchangeably because of no modification to the ROM solution algorithm. The reconstruction operation also bypasses an expensive Galerkin or Petrov-Galerkin projection by using any regression model such as Gaussian process, radial basis function regression, or artificial neural networks to map between $\varvec{\mu }$ and reduced manifolds.

This study utilizes a non-intrusive ROM approach because it does not require any cumbersome modifications of FOM source codes^27,28^ and can be applied to any physics-based problems easily. Additionally, the non-intrusive ROM proposed by Kadeethum et al.^29,30^ illustrates its capacity to handle high-dimensional input, which is extremely difficult for POD-based ROM due to its dependence on a high dimensional reduced basis (i.e., high Kolmogorov n-width)^29^. Another advantage of non-intrusive ROMs for coupled multiphysics processes (i.e., many primary variables) is to selectively focus on the quantities of interest^31^. For instance, if we are interested in the saturation field of the two-phase flow problems, we can build our non-intrusive ROM for the saturation field without necessarily constructing the ROM for pressure and velocity fields, which could save substantial computational resources.

Even though ROM can deliver an acceptable accuracy with a much lower computational cost, it might not be suitable for an application in which precision is paramount. In such cases, FOM is still preferable. As mentioned previously, FOM’s solver can take a significant amount of computational resources, especially if the system is nonlinear. Besides, a solver of nonlinear PDEs relies heavily on the initial guess, preconditioner, or solving algorithm^32–35^. With a non-optimal initial guess, the solver might not converge at all. Hence, using machine learning to assist this solver (i.e., by speed up or improved convergence) could alleviate this. Recently, the use of machine learning to enhance, accelerate, or assist FOMs’ performance has been proposed. Some examples include (1) improving the efficacy of FOMs’ solver^36–39^, (2) guiding dynamic mesh refinement^40^, or (3) fine-tuning stabilization parameters^41,42^. Besides, there have also been other endeavors to speed up or stabilize a nonlinear solver; for instance, using data-driven modeling to accelerate pressure projection inside multi-grid solver^43^, residual smoothing, which aims to smooth any sharp gradients of Newton iterative steps^44^, or Krylov subspace algorithm, which aims to uses a low-rank least-squares analysis to search for equilibrium state of all degrees of freedom^45^. This paper proposes initializing FOM’s nonlinear solver by using ROM’s prediction. We hypothesize that as ROM’s prediction becomes closer to the real or converged solution of FOM, the nonlinear solver would require fewer iterations to converge compared to a conventional method of choosing an initial guess, resulting in a lower computational cost. Hereafter, we will refer to this technique as ROM-assisted FOM. We note that this approach is in line with using optimization algorithms such as genetic algorithm^46^, Powell’s method^47^, or chaos optimization algorithm^48^ to locate an optimal location of an initial guess.

The rest of the manuscript is summarized as follows. In “Method” section, we outline our proposed framework as well as how to use different ROMs to handle different types of $\varvec{\mu }$. We present our results through four main examples, which represent different physics as well as numerical methods (e.g., finite volume, finite element, or hybrid methods). Moreover, we also show that our proposed approach could handle both homogeneous and heterogeneous $\varvec{\mu }$, followed by the discussion of the reduction of the computational cost with respect to different ROMs. We also summarize our findings in “Conclusion”. In the supplementary information, we describe the governing equations, problem setting, and solution method for each main example in detail. Furthermore, details of all ROMs (both intrusive and non-intrusive approaches) used in this study are presented in the supplementary information.

## Methods

**Figure 1:**
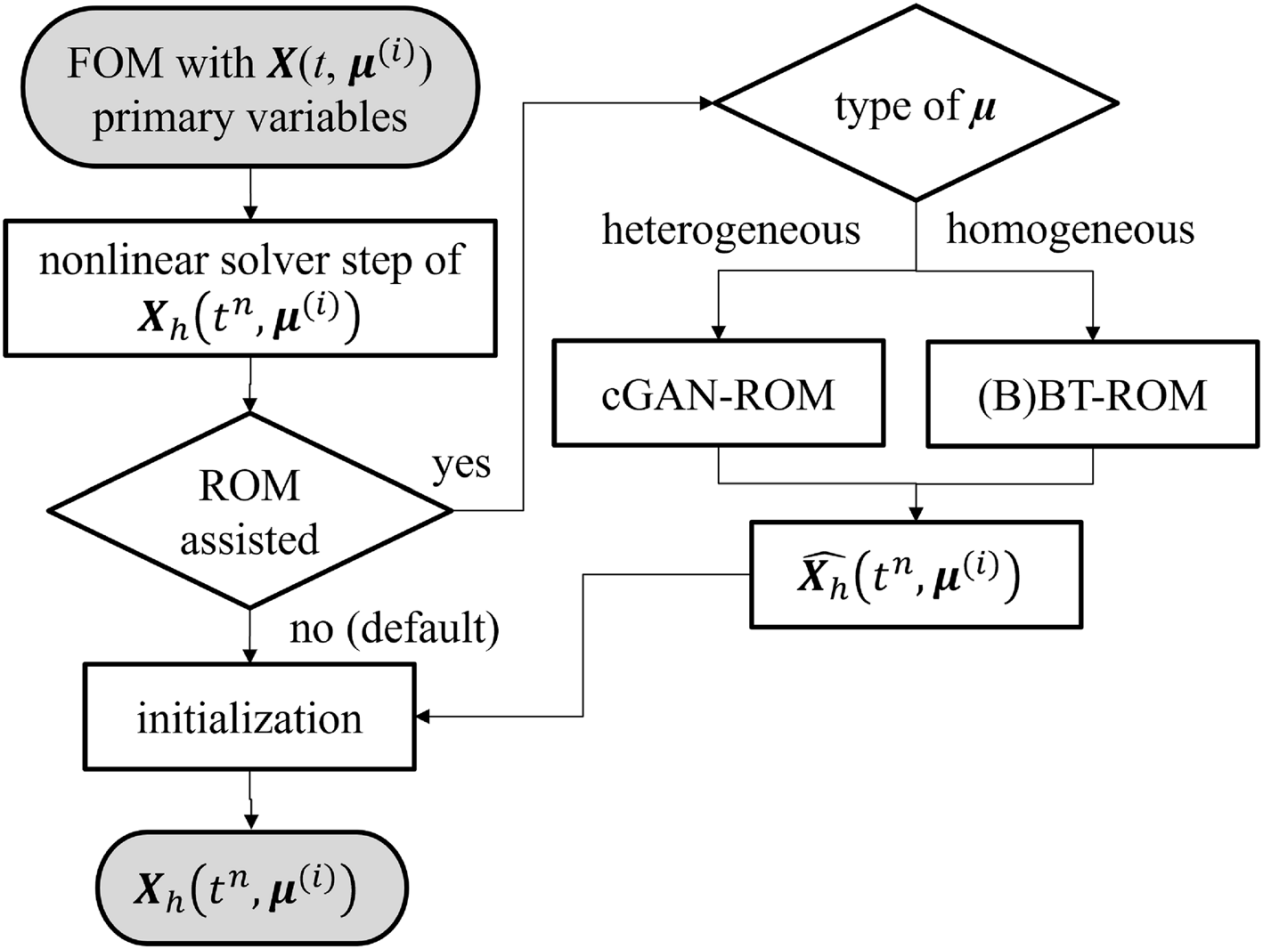
FOM nonlinear solver with ROM assisted procedures. We note that $\varvec{X}$ is an exact solution, $\varvec{X}_h$ is quantities of interest obtaining from FOM, and $\widehat{\varvec{X}}_h$ is an approximation of $\varvec{X}_h$ obtaining from ROM. $\varvec{\mu }$ is a set of parameterized parameters, which could be homogeneous or heterogeneous parameter.

The summary of our proposed procedures is shown in Fig. 1. Here, we have a system of parameterized PDEs as

1 $$\begin{aligned} \begin{aligned} \varvec{F}\left( t, \varvec{\mu }\right)&= \varvec{0} \, \text{ in } \, \Omega , \\ \varvec{X}&=\varvec{f}_{D} \,\text { on }\, \partial \Omega _{D},\\ - \nabla \varvec{X} \cdot \mathbf {n}&=\varvec{f}_N \, \text{ on } \, \partial \Omega _{N}. \\ \varvec{X}&=\varvec{X}_{0} \,\text { in }\, \Omega \text{ at } t^n = 0, \end{aligned} \end{aligned}$$

where $\varvec{F}\left( \cdot \right)$ corresponds to the system of time dependent PDEs, $\Omega \subset \mathbb {R}^{n_d}$ (${n_d} \in \{1,2,3\}$) denotes the computational domain, $\partial \Omega _{D}$ and $\partial \Omega _{N}$ denote the Dirichlet and Neumann boundaries, respectively. $\varvec{f}_{D}$ and $\varvec{f}_N$ are prescribed values on $\partial \Omega _{D}$ and $\partial \Omega _{N}$, respectively. $\varvec{X}_{0}$ is an initial value of $\varvec{X}$. The time domain $\mathbb {T} = \left( 0, \tau \right]$ is partitioned into $N^t$ subintervals such that $0=: t^{0}<t^{1}<\cdots <t^{N} := \tau$, We denote $t^{n} \in \mathbb {T}$ as $n{th}$ time-step, $n\in [0,N]$. $\varvec{X}$ is the primary variable. The parameter domain $\mathbb {P}$ is discretized by means of $\mathrm {M}$ realizations, i.e., $\varvec{\mu }^{(1)}$, $\varvec{\mu }^{(2)}$, $\dots$, $\varvec{\mu }^{(\mathrm {M-1})}$, $\varvec{\mu }^{(\mathrm {M})}$, and $\varvec{\mu }^{(i)}$ represents $i{th}$ member, where $i\in [0,\mathrm {M}]$. In general, $\varvec{\mu }$ could correspond to physical properties, geometric characteristics, or boundary conditions, and $\varvec{\mu }$ could be either a homogeneous or heterogeneous variable. Here, $\varvec{X}$ is an exact solution of $\varvec{F}\left( \varvec{X}; t, \varvec{\mu }\right)$, and ${\varvec{X}_h}$ is an approximation of $\varvec{X}$ obtained from FOM.

Since every nonlinear solver step could be considered computationally expensive, we aim to reduce this computational cost by using ROM prediction ($\widehat{\varvec{X}}_h$) as an initial guess (ROM-assisted) for the nonlinear solver. Our rationale is as $\widehat{\varvec{X}}_h$ getting closer to ${\varvec{X}_h}$, the nonlinear solver will take less iterations to step toward converged solutions. Note that $\widehat{\varvec{X}}_h$ is an approximation of ${\varvec{X}_h}$ delivered by ROM. We note that instead of using $\widehat{\varvec{X}}_h$ approximated from ROM, one could use $\widehat{\varvec{X}}_h$ obtained through a solution of a linearized version of nonlinear PDEs as an initial guess. This technique has been successfully applied to solve Navier-Stokes^49^ and magnetohydrodynamic^50^ equations using a solution of Stokes equations as an initial guess.

Our procedures are as follows: at every solving step of FOM with parameters $t^n, \varvec{\mu }^{(i)}$ for a transient problem or $\varvec{\mu }^{(i)}$ for a steady-state problem, we will use a trained ROM-assisted or initial guess for the nonlinear solver (i.e., we use $\widehat{\varvec{X}}_h\left( t^n, \varvec{\mu }^{(i)}\right)$ or $\widehat{\varvec{X}}_h\left( \varvec{\mu }^{(i)} \right)$ as an initial guess for solving ${\varvec{X}_h}\left( t^n, \varvec{\mu }^{(i)} \right)$ or ${\varvec{X}_h}\left( \varvec{\mu }^{(i)} \right)$). We note that as a default one might use $\varvec{0}$ as an initial guess for solving ${\varvec{X}_h}\left( \varvec{\mu }^{(i)} \right)$ or ${\varvec{X}_h}\left( t^{n-1}, \varvec{\mu }^{(i)} \right)$ as an initial guess for ${\varvec{X}_h}\left( t^n, \varvec{\mu }^{(i)}\right)$. We note that, for time-dependent problems, one could also use an extrapolation of a polynomial regression of ${\varvec{X}_h}\left( t^{n-1}, \varvec{\mu }^{(i)} \right)$, ${\varvec{X}_h}\left( t^{n-2}, \varvec{\mu }^{(i)} \right)$, $\cdots$, ${\varvec{X}_h}\left( t^{1}, \varvec{\mu }^{(i)} \right)$, $\varvec{0}$ as an initial guess^51^. To clarify, if one uses a polynomial regression of ${\varvec{X}_h}\left( t^{n-1}, \varvec{\mu }^{(i)} \right)$ and ${\varvec{X}_h}\left( t^{n-2}, \varvec{\mu }^{(i)} \right)$, we have a linear extrapolation, and a polynomial regression of ${\varvec{X}_h}\left( t^{n-1}, \varvec{\mu }^{(i)} \right)$, ${\varvec{X}_h}\left( t^{n-2}, \varvec{\mu }^{(i)} \right)$, and ${\varvec{X}_h}\left( t^{n-3}, \varvec{\mu }^{(i)} \right)$ would represent a quadratic extrapolation.

In this study, we generalize $\varvec{\mu }$ as either a homogeneous or heterogeneous parameter. For a heterogeneous parameter, the parameter is varied throughout a computational domain $\Omega$, but it is not altered through time (i.e., $\varvec{\mu }(x, y)$ for a two-dimensional domain). The ROM used to tackle a heterogeneous $\varvec{\mu }$ is cGAN-ROM, which is discussed in Supplementary sec. 5.1. In short, the cGAN-ROM takes a heterogeneous $\varvec{\mu }$ as its input and delivers quantities of interest, $\widehat{\varvec{X}_h}$. For a homogeneous parameter, the parameter is constant throughout a computational domain $\Omega$ as well as the time domain $\mathbb {T}$. We use either BT-ROM (see Supplementary sec. 5.2) or BBT-ROM (see Supplementary sec. 5.3) to handle this type of $\varvec{\mu }$. The main difference between BT-ROM and BBT-ROM is we apply a boosting algorithm to enhance the BT-ROM^25^ performance and help it combat imbalanced data set. We propose this model because the primary challenge for applying machine learning techniques to the physics-based problems with a point source (or Dirac delta distribution); for instance, contact problems or subsurface flow with wells is that there might be a very small part of a domain that is altered while the majority of it remains constant^52,53^. All ROMs used in this study are data-driven, or in other words, non-intrusive. Hence, the procedures proposed here, Fig. 1, are easily applied to other nonlinear physics-based problems, which rely on traditional nonlinear solvers (i.e., Picard’s or Newton’s iteration).

## Results

### Data generation and model selection

Our proposed approach is illustrated in the following sections through four types of physics-based nonlinear problems. We have summarized some key points, including our findings, in Table 1. We will discuss each point in the table in detail throughout the subsequent sections. We want to emphasize that, throughout this study, we generalize $\varvec{\mu }$ as either a homogeneous or heterogeneous parameter. When heterogeneous (Example 1), the parameters are a function of space, i.e., $\varvec{\mu }(x, y)$ for a two-dimensional domain. When homogeneous (Examples 2-4), the parameter $\varvec{\mu }$ does not depend on space. For all four examples, the parameters are time-independent, i.e., they are not altered as time progresses. The first problem is the steady-state Richards’ equation^54,55^ with a heterogeneous material ($\varvec{\kappa }(x, y)$), which represents a water flow in unsaturated soils (Supplementary sec. 1). This equation is well-known for a nonlinear profile of the water head due to the relative permeability coefficient. With heterogeneous permeability fields, nonlinearity becomes a multi-dimensional problem. In this case, we employ reduced order modeling using conditional generative adversarial networks (cGAN-ROM) (Supplementary sec. 5.1) because it is suitable to handle high-dimensional (i.e., spatially distributed) $\varvec{\mu }$. The second problem is a contact problem over a hyperelastic material, which has a wide range of applications from subsurface energy storage to indentation problems in biomedical and material engineering (Supplementary sec. 2)^56–58^. The nonlinearity of this problem arises from two parts. The first one is caused by the material property that allows the large deformation problem. The second part is due to enforcing contact constraints. We test reduced order modeling with Barlow Twins (BT-ROM) (Supplementary sec. 5.2), reduced order modeling with boosting Barlow Twins (BBT-ROM) (Supplementary sec. 5.3), and intrusive reduced order modeling through Galerkin projection (in-ROM) (Supplementary sec. 5.4) because we have a homogeneous $\varvec{\mu }$.

The third problem is two-phase flow in a layered porous material (Supplementary sec. 3), which is applicable to subsurface energy recovery, environmental remediation, or $\mathrm {CO_2}$ sequestration^59–61^. Example 3 is a time-dependent problem with a homogeneous $\varvec{\mu }$ in contrast to steady-state problems in the first and the second examples. Example 3 is a well-known nonlinear PDE; the nonlinearity is caused by relative permeability, capillary pressure, and an interplay between pressure and saturation. We only use BBT-ROM (Supplementary sec. 5.3) because of its superior performance shown in Example 2. The last problem, Example 4, shows our framework applicability in enhancing fracture propagation modeling through the phase-field approach^62,63^. This problem can be applied to a material and environmental science^64,65^. Even though we are working with a linear elasticity scheme, the nonlinearity is caused by the energy constraint used to mimic fracture propagation. We represent the discontinuity feature with the continuous phase field function. Since we have a time-dependent problem with a homogeneous $\varvec{\mu }$ as in Example 3, we again apply BBT-ROM (Supplementary sec. 5.3). We have summarized each example’s nonlinear solver and how we initialize them in Table 2.

**Table 1:** Summary of main information for each example.

Example		1	2.1	2.2	2.3	3	4
Degrees of freedom		$\left[ 128 \times 128\right]$	3993	3993	70602	2548	5823
Parameters $\varvec{\mu }$		$\varvec{\kappa }(x, y)$	$\nu , \mathrm {In_{D}}$	$\mathrm {In_{R}}, \mathrm {In_{D}}$	*x*, *y*	$t, \varvec{\kappa }_{\mathrm {top}}$	$t, \mathrm {F}$
Training set ($\mathrm {M_{train}}$)		9000	1600	1600	1600	13600 $(N_t \mathrm {M_{train}})$	4509 $(N_t \mathrm {M_{train}})$
Validation set ($\mathrm {M_{validation}}$)		500	$5\%$ of $\mathrm {M_{train}}$	$5\%$ $\mathrm {M_{train}}$	$5\%$ $\mathrm {M_{train}}$	$5\%$ $N_t \mathrm {M_{train}}$	$5\%$ $N_t \mathrm {M_{train}}$
Testing set ($\mathrm {M_{test}}$)		500	100	100	100	1900 ($N_t \mathrm {M_{test}}$)	1002 ($N_t \mathrm {M_{test}}$)
Training time (h)	cGAN-ROM	4.00	$\times$	$\times$	$\times$	$\times$	$\times$
	BT-ROM	$\times$	0.67	0.67	1.0	$\times$	$\times$
	BBT-ROM	$\times$	0.50	0.50	0.92	0.75	0.45
	in-ROM	$\times$	1.00	1.00	1.83	$\times$	$\times$
Number of nonlinear iterations (–)	Default init.	15.04	6.10	6.30	6.16	934.68	6986.00
	cGAN-ROM	4.12	$\times$	$\times$	$\times$	$\times$	$\times$
	BT-ROM	$\times$	neg.	neg.	5.40	$\times$	$\times$
	BBT-ROM	$\times$	2.62	3.04	4.76	758.57	3412.00
	in-ROM	$\times$	1.09	1.08	neg.	$\times$	$\times$
Speed up (%)	cGAN-ROM	72.63	$\times$	$\times$	$\times$	$\times$	$\times$
	BT-ROM	$\times$	neg.	neg.	12.33	$\times$	$\times$
	BBT-ROM	$\times$	57.05	51.75	22.72	18.84	49.00
	in-ROM	$\times$	82.12	82.86	neg.	$\times$	$\times$

Example 1 has heterogeneous parameters, and its FOM relies on structured grids - $\mathrm {DOF} = [\mathrm {DOF}_x,\mathrm {DOF}_y]$. Examples 2, 3, and 4 have homogeneous parameters, and their FOMs use unstructured meshes. Speed up is calculated by the difference between a number of nonlinear iterations using ROM-assisted and default initialization, then divided by a number of nonlinear iterations of default initialization. neg. represents a case where using ROM-assisted causes an incremental cost (i.e., negative affect), default init. is shorted for default initialization, and $\times$ represents not applicable. The prediction cost of in-ROM is much higher than the rest, which also affects the actual speed up. We discuss this effect on the actual cost saving in Example 2. Since we observe a better as well as stable performance of BBT-ROM in Example 2, we only apply BBT-ROM to Examples 3 and 4. For steady-state problems (Examples 1 and 2), we have a training set of $\mathrm {M_{train}}$. For transient problems (Examples 3 and 4),we have a training set of $N_t \mathrm {M_{train}}$. The same goes with validation and testing sets.

**Table 2:** Summary of each example’s nonlinear solver scheme and initialization.

	Example 1	Example 2	Example 3	Example 4
Nonlinear solver	Newton iteration	PETSc SNES	Picard iteration	Newton iteration
Linear solver	Direct solver	MUMPS	GMRES with multigrid	Direct solver
Time characteristic	Steady-state	Steady-state	Transient	Transient
Default initialization	$\varvec{0}$	$\varvec{0}$	${\varvec{X}_h}\left( t^{n-1}, \varvec{\mu }^{(i)} \right)$	${\varvec{X}_h}\left( t^{n-1}, \varvec{\mu }^{(i)} \right)$
ROM-assisted	$\widehat{\varvec{X}}_h\left( \varvec{\mu }^{(i)} \right)$	$\widehat{\varvec{X}}_h\left( \varvec{\mu }^{(i)} \right)$	$\widehat{\varvec{X}}_h\left( t^n, \varvec{\mu }^{(i)}\right)$	$\widehat{\varvec{X}}_h\left( t^n, \varvec{\mu }^{(i)}\right)$

### Example 1: Richards’ equation with heterogeneous material

Example 1 focuses on steady-state Richards’ equation with a heterogeneous material; see Supplementary sec. 1 for more details of the problem statement and governing equations. We aim to study the impact of ROM initialization (ROM-assisted) on nonlinear solver iterations. The numerical solution of Richards’ equation is challenging due to its nonlinearity as well as complexity in relative conductivity and capillary pressure relations^54^. To introduce further complexities, a heterogeneity in subsurface structures could cause a sharp discontinuity resulting in difficulties in solving such a nonlinear system. Here, we use a data-driven framework, cGAN-ROM—Supplementary sec. 5.1^29^, to speed up a nonlinear solver used for solving Richards’ equation.

**Figure 2:**
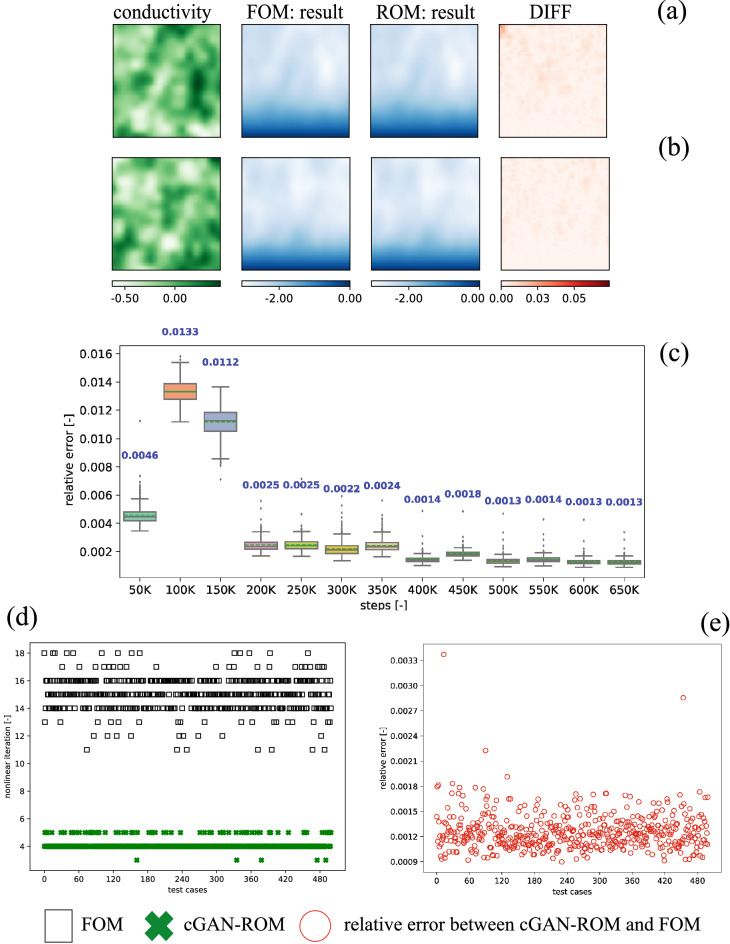
Example 1—results: (**a**,**b**) are samples of test case results. We have an heterogeneous conductivity as a parameter $\varvec{\mu }$. The FOM and ROM results shown here are pressure head. Both conductivity field and pressure head shown here are dimensionless (see Supplementary sec. 1). (**c**) relative error results of validation set as a function of training steps—we note that each step refers to each time we perform back-propagation, including updating both generator and discriminator’s parameters. The blue text represents a mean value. (**d**) Number of nonlinear iterations: using FOM default initialization, zero-vector initialization, (black square) and using cGAN-ROM-assisted (green cross) and (**e**) relative error of test dataset (red dot). The relative error (see Eq. 3) is calculated between FOM with default initialization (black square) and cGAN-ROM (green cross).

We solve the Richards’ equation in a dimensionless setting, see Supplementary sec. 1. The parameters $\varvec{\mu }$ in this example are the heterogeneous conductivity fields $\kappa$. The $\kappa$ is generated using Normal prior with mean $\log (\kappa )$ of 0.0, the $\log (\kappa )$ standard deviation of 0.25, and the correlation length is 10. The state variable or output here is pressure head, which represents a height of a water column inside a well. The nonlinear discretized equations are solved using the “nlsolve” function from a standard Julia nonlinear solver package^66^ with the default settings (i.e., convergence tolerance of $1 \times 10^{-8}$), which uses a Newton iteration with a trust region. We employ a training set $\mathrm {M_{train}} = 9000$, validation set $\mathrm {M_{validation}} = 500$, and test set $\mathrm {M_{test}} = 500$. Our results are presented in Fig. 2. In this figure, (a) and (b) are samples of test dataset results (randomly selected 2 out of 500 test cases). From these two figures we can see that, our model can provide reasonable approximations of the FOM results. The point-wise difference between solutions produced by the FOM and ROM (further referred to as DIFF) is calculated by

2 $$\begin{aligned} {\mathrm{DIFF}}(\varvec{X})= \left| \varvec{X}_h - \widehat{\varvec{X}}_h\right| . \end{aligned}$$

Here, $\varvec{X}_h$ is a FOM solution, and $\widehat{\varvec{X}}_h$ is an approximation of $\varvec{X}_h$ produced by the ROM. Judging from Fig. 2a,b, the DIFF values are relatively small.

This FOM employs a $128 \times 128$ structured mesh, therefore the number of degrees of freedom is 16,384. A number of nonlinear iterations are presented in Fig. 2d. We summarize our finding in Table 1. On average, using the FOM’s default initialization, i.e. zero-vector initialization, the nonlinear solver takes 15.04 iterations, while the nonlinear solver with cGAN-ROM-assisted takes 4.12 iterations. These comparisons illustrate that using the cGAN-ROM-assisted decreases a number of nonlinear iterations (speed up) by 72.63%. For a wall time comparison, for each test case using Intel(R) Core(TM) i9-9960X, solving a steady-state Richards’ equation using FOM default initialization requires about 3.89 s while using cGAN-ROM-assisted each test case takes, on average, approximately 2.06 s. We note that each cGAN-ROM prediction takes only 0.001 s, which is insignificant compared to the FOM solver. Hence, the cGAN-ROM-assisted speeds up a calculation by 46.98%. The training time of the cGAN-ROM through NVIDIA Quadro RTX 5000 Mobile Max-Q is about 4 h (650,000 steps).

The relative error results are presented in Fig. 2c for the validation set and Fig. 2e for the test set. The relative error is calculated by

3 $$\begin{aligned} \mathrm {relative} \ \mathrm {error} = \frac{|| \varvec{X}_h - \widehat{\varvec{X}}_h||}{|| \varvec{X}_h||}, \end{aligned}$$

where $||\cdot ||$ denotes the $L^2$ norm. From Fig. 2c in general, we observe that as the training progresses (i.e., larger steps), the relative error is reduced. We picked the model at the 650,000th step for the test set, as it performs the best (average relative error of 0.13%) against the validation set. From Fig. 2e, the model delivers approximately the same level of accuracy as it performs for the validation set (i.e., the average relative error is about 0.13%).

### Example 2: Contact problems with hyperelastic material

**Figure 3:**
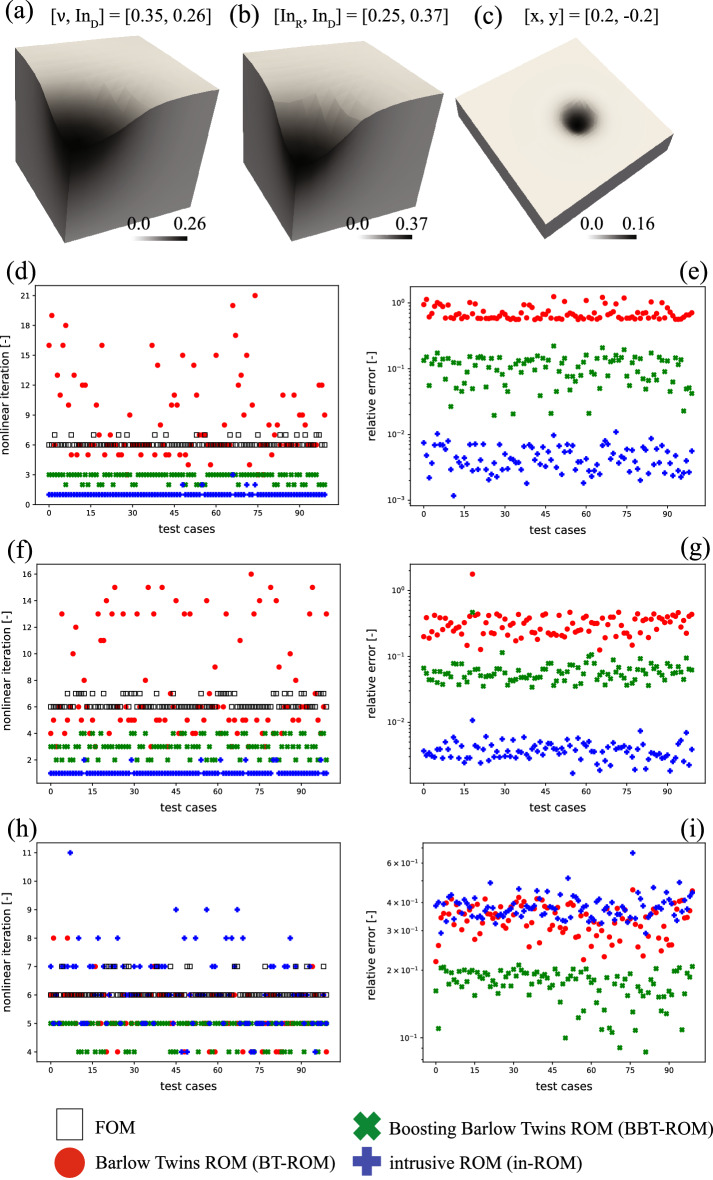
Example 2 - results: samples of test dataset results where (**a**) $\nu$ and $\mathrm {In_{D}}$ are parameters, (**b**) $\mathrm {In_{R}}$ and $\mathrm {In_{D}}$ are parameters, and (**c**) indentation location ($\left( x,y \right)$) is a parameter. (**d**) Number of nonlinear iterations and (**e**) relative error of test dataset results where $\nu$ and $\mathrm {In_{D}}$ are parameters, (**f**) number of nonlinear iterations and (**g**) relative error of test dataset results where $\mathrm {In_{R}}$ and $\mathrm {In_{D}}$ are parameters, and (**h**) number of nonlinear iterations and (**i**) relative error of test dataset results where indentation location ($\left( x,y \right)$) is a parameter. The nonlinear solver iteration using default FOM initialization, zero-vector initialization, is shown with a black square, BT-ROM-assisted is shown with a red dot, BBT-ROM-assisted is illustrated with a green cross, and in-ROM-assisted is presented with a blue plus. The relative error (see Eq. 3) is calculated between each ROM (BT-ROM (red dot), BBT-ROM (green cross), or in-ROM (blue plus) and FOM (black square).

Example 2 focuses on steady-state contact problems, where a rigid spherical indenter achieves frictionless contact on a hyperelastic domain. Details of problem statement and governing equation can be found in Supplementary sec. 2. Similar to Example 1, we aim to investigate an effect of ROM-assisted on nonlinear solver iterations. However, unlike the previous example, the media is homogeneous, but the parameters or $\varvec{\mu }$ could take their values in a certain range. We use the model developed by Kadeethum et al.^25^, see Supplementary sec. 5.2, and its improved version discussed in Supplementary sec. 5.3 as our ROM. Throughout this example, we have three test scenarios corresponding to using (1) Poisson’s ratio and indentation depth as parameters—$\varvec{\mu } = [\nu , \mathrm {In_{D}}]$ (see Fig. 3a), (2) indentation radius and indentation depth as parameters - $\varvec{\mu } = [\mathrm {In_{R}}, \mathrm {In_{D}}]$ (see Fig. 3b), and (3) indentation location is a parameter - $\varvec{\mu } = [x, y]$ (see Fig. 3c). For all three scenarios, we have a training set $\mathrm {M_{train}} = 1600$, validation set $\mathrm {M_{validation}} = 5\%$ of $\mathrm {M_{train}}$ (randomly selected—see Supplementary sec. 5.2 and Supplementary sec. 5.3 for more detail), and test set $\mathrm {M_{test}} = 100$.

Throughout Example 2, we will compare a number of nonlinear iterations used to solve each test case (i.e., different values of $\varvec{\mu }$). We illustrate the impacts of using ROM to initialize the nonlinear solver (i.e., initial guess). We use three types of ROMs; (1) BT-ROM—see Supplementary sec. 5.2, (2) BBT-ROM—see Supplementary sec. 5.3, and (3) in-ROM—see Supplementary sec. 5.4. For BBT-ROM, we use $\mathrm {N_{en}}=5$ and a sub-sample of a quarter of the total training data. We use PETSc SNES as a nonlinear solver and MUMPS as a linear solver^67^, and set absolute and relative tolerances of $1 \times 10^{-6}$ and $1 \times 10^{-16}$, respectively. We utilize a backtracking line search with slope descent parameter of $1 \times 10^{-4}$, initial step length of 1.0, and quadratic order of the approximation. To present our findings, we focus on two quantities: (1) a number of nonlinear iterations in which we use zero-vector initialization, or FOM default initialization, as a base case; and subsequently, compare the base case with a number of nonlinear iterations using predictions from BT-ROM-, BBT-ROM-, or in-ROM-assisted FOM and (2) relative error calculated by Eq. (3) (i.e., how accurate each ROM model is at mimicking FOM results).

#### Example 2.1: Young’s modulus and indentation depth are parameters

We use $\varvec{\mu } = [\nu , \mathrm {In_{D}}] \in (0.1, 0.4) \times (0.1, 0.3)$, and the details of the model settings can be found in Supplementary sec. 2.1. An unstructured mesh is used for this FOM, with 3993 degrees of freedom, which means there are 1331 degrees of freedom for each displacement in *x*-, *y*-, and *z*-directions. One example of the test dataset is presented in Fig. 3a. A number of nonlinear iterations are presented in Fig. 3d. For results of a number of nonlinear iterations, on average, using FOM default initialization, zero-vector initialization, the nonlinear solver takes 6.10 iterations. Using BT-ROM-, BBT-ROM-, and in-ROM-assisted, the nonlinear solver takes 7.93, 2.62, and 1.09, respectively. These results imply that using BT-ROM-assisted increases the computational burden to the system by 30.00%. However, using BBT-ROM- or in-ROM-assisted, we achieve speed up by 57.05% or 82.12%, respectively.

We note that one nonlinear iteration takes approximately 10 s (61 s for 6.10 iterations) computed using Intel(R) Core(TM) i7-9750H CPU. As a result, the BBT-ROM model saves approximately 35 s per FOM evaluation, and the in-ROM saves approximately 50 s per FOM evaluation. The prediction time of the BBT-ROM model takes about 0.001 s, while the in-ROM takes around 8 s (both using the same Intel(R) Core(TM) i7-9750H CPU). Hence, the actual wall time saving for the in-ROM is 42 s. The training time of the BBT-ROM through NVIDIA Quadro RTX 5000 Mobile Max-Q is about 30 min. The training time of the in-ROM using Intel(R) Core(TM) i7-9750H CPU is about 60 min. We disregard discussion of the BT-ROM because it increases the computational burden (i.e., using BT-ROM-assisted, the nonlinear solver takes more iterations to coverage).

The relative error results are presented in Fig. 3e. We observe that the error of the BT-ROM is significantly higher than those of the BBT-ROM or in-ROM. This could be explained by the fact that the primary challenge for applying machine learning techniques to the contact problem is how to deal with imbalanced training data^52,53^. To elaborate, as we have only one point of contact, there is only a small area where the deformation occurs while most of the domain remains undeformed. In BBT-ROM we solve this problem by applying a boosting technique to the BT-ROM (see Supplementary sec. 5.3). Consequently, the relative error of the BBT-ROM is one to two orders of magnitude less than that of the BT-ROM. The in-ROM performs the best in this setting with around 0.1% relative error.

We observe correlations between a number of nonlinear iterations and relative error. As the accuracy of the initial guess increases, a number of nonlinear iterations decrease. This situation happens because as the initial guess is closer to an actual solution (FOM solution), fewer iterations of the nonlinear solver are required to reach convergence.

#### Example 2.2: Indentation radius and indentation depth are parameters

Next, we use $\varvec{\mu } = [\mathrm {In_{R}}, \mathrm {In_{D}}] \in (0.15, 0.4) \times (0.1, 0.4)$, and the details of model settings could be found in Supplementary sec. 2.2. We present one of the test dataset in Fig. 3b and a number of nonlinear iterations used by the nonlinear solver in Fig. 3f. From Fig. 3f, on average, using FOM default initialization, the solver takes 6.30 iterations. Using BT-ROM-, BBT-ROM-, and in-ROM-assisted, the solver takes 7.52, 3.04, and 1.08 iterations, respectively. The trend of the number of iterations is in line with the previous example, i.e., using BT-ROM-assisted increases the computational burden to the system by 19.37%, while using BBT-ROM- or in-ROM-assisted achieves speed up by 51.75% or 82.86%.

Similarly to the previous example (the number of degrees of freedom is identical - the total degrees of freedom for this FOM, an unstructured mesh, is 3993, which means 1331 for each displacement in x-, y-, and z-directions.), one nonlinear iteration takes approximately 10 s (63 s for 6.30 iterations) computed through Intel(R) Core(TM) i7-9750H CPU. Consequently, the BBT-ROM model saves us around 33 s per FOM evaluation, and the in-ROM saves us approximately 52 s per FOM evaluation. The prediction time of the BBT-ROM model takes about 0.001 s, while the in-ROM takes around 8 s (both using the same Intel(R) Core(TM) i7-9750H CPU). Hence, the actual wall time saving for the in-ROM is 44 s. Again, the training time of the BBT-ROM through NVIDIA Quadro RTX 5000 Mobile Max-Q is about 30 min. The training time of the in-ROM using Intel(R) Core(TM) i7-9750H CPU is about 60 min.

In line with the previous example, we observe correlations between nonlinear iterations and relative error. To elaborate, a ROM that provides a more accurate prediction can assist in reducing a number of nonlinear iterations (i.e., as an initial guess is closer to a FOM solution, the less iteration the nonlinear solver requires to take to converge.). The relative error results are presented in Fig. 3g. We observe that the in-ROM delivers the most accurate predictions (a relative error on average of 0.1%), the BBT-ROM comes in second (a relative error on average of 6.0%), and the BT-ROM has the worst accuracy (a relative error on average of 32.07%). The challenge of contact problems, similar to the previous one, also stems from the fact that we have only a small area of contact.

#### Example 2.3: Indentation location is a parameter

Lastly, we use the indentation location as our parameter, $\varvec{\mu } = [x, y] \in (-0.3, 0.3) \times (-0.3, 0.3)$. The details of this setting are presented in Supplementary sec. 2.3. The number of degrees of freedom for this FOM is 70602, an unstructured mesh, which means 23,534 for each displacement in x-, y-, and z-directions. We present one of the test dataset in Fig. 3c and a number of nonlinear iterations in Fig. 3h. On average, using zero-vector initialization, the nonlinear solver takes 6.16 iterations, using BT-ROM-, BBT-ROM-, and in-ROM-assisted, the solver takes 5.4, 4.76, and 6.22 iterations, respectively. These results are different from the previous two settings, as using in-ROM, the computational cost is increased by 0.97%. BT-ROM achieves speed up by 12.33%, and BBT-ROM decreases the computational cost by 22.72%.

This setting has many more degrees of freedom than the previous two settings resulting in one nonlinear iteration computed through Intel(R) Core(TM) i7-9750H CPU taking approximately 35 s (or 215.6 s for 6.16 iterations). In short, using the in-ROM-assisted takes around 2.10 s more than the FOM default initialization (zero-vector initialization). In contrast, compared with the zero-vector initialization, using the BT-ROM, the solver takes 26.60 s less, and using the BBT-ROM, the nonlinear solver takes 49.00 s less. The prediction time of the BT-ROM and BBT-ROM models is about 0.001 s (similar to two previous examples). The BT-ROM and BBT-ROM training times are approximately 60 and 55 min computed using NVIDIA Quadro RTX 5000 Mobile Max-Q, respectively. It is higher than the two previous examples because the number of degrees of freedom is substantially larger. The in-ROM takes 11 s per prediction resulting in the actual wall time incremental of 13.10 s. The training time of the in-ROM using Intel(R) Core(TM) i7-9750H CPU is about 110 min.

Similar to two previous settings, there are correlations between a number of nonlinear iterations and relative error, see Fig. 3h-i. To elaborate, we observe that the higher accuracy ROMs provide fewer nonlinear iterations (i.e., the nonlinear solver required to converge). As an initial guess is closer to a FOM solution, it is easier for the nonlinear solver to step toward converged solutions. The relative error results of this setting are presented in Fig. 3i. We observe that the BBT-ROM offers the most accurate prediction and has a relative error on average of 17.17%. The BT-ROM comes the second and has a relative error on average of 33.67%. In contrast to the two previous settings, the in-ROM has the lowest accuracy with a relative error on average of 38.37%. We speculate that as the in-ROM relies on POD (linear manifolds), it fails to capture this setting as the problem lies within nonlinear manifolds. Please refer to Kadeethum et al.^13,25^ for detailed discussions on linear and nonlinear manifolds. Furthermore, the BT models (BT-ROM and BBT-ROM) outperformed the in-ROM. In line with two previous settings, we still have only a small area of contact (imbalanced data problem), resulting in BBT-ROM outperforming BT-ROM.

### Example 3: Two-phase flow in layered porous material

Throughout Example 3, we focus on a *time-dependent* two-phase flow in layered porous media. The details of the problem statement and governing equation can be found in Supplementary sec. 3. In this example, we have two state variables, pressure ($p_h$) and saturation ($s_h$), and both variables have the same number of degrees of freedom of 2548. Similar to Example 2, our $\varvec{\mu }$ is homogeneous and takes values in a certain range. Hence, BT-ROM (Supplementary sec. 5.2) and BBT-ROM (Supplementary sec. 5.3) are suitable in this case. As we have illustrated in Example 2, BBT-ROM performs better than BT-ROM (as well as more stably than in-ROM (Supplementary sec. 5.4)), we use only BBT-ROM with $\mathrm {N_{en}}=10$ and sub-sample of a quarter of the total training data in this example. We use $\varvec{\mu } = [t, \varvec{\kappa }_{\mathrm {top}}]\in (0.0, 100.0) \times (1.08 \times 10^{-11}, 9.97 \times 10^{-10})]$. Note that our model treats the time domain as one of the parameters^13,25^. We fix the permeability of the bottom layer and set the porosity in the top and bottom layers to 0.1 and 0.2, respectively. We inject one phase on the left and produce both phases on the right by imposing a fixed pressure on both boundaries. The top and bottom boundaries are closed to flow. The viscosity and density of the injected and displaced phases are ($1.0 \times 10^{-3}$ ${\mathrm{Pa}} \, {\mathrm{s}}$, $1.0 \times 10^{3}$ ${\mathrm{kg}}/{\mathrm{m}}^{3}$) and ($5.0 \times 10^{-3}$ ${\mathrm{Pa}} \,{\mathrm{s}}$, $7.0 \times 10^{2}$ ${\mathrm{kg}}/{\mathrm{m}}^3$), respectively. It is worth mentioning that for defining $\varvec{\mu }$ in this example, we use the permeability divided by the viscosity of the injected phase. We have a training set $\mathrm {M_{train}} = 136$ resulting in $N_t \mathrm {M_{train}} =$ 13,600. To elaborate, we have a training set of $\mathrm {M_{train}}$ generated by choosing 136 values of parameters and, for each parameter set, we collect solutions for 100 timestamps. We select a validation set $N_t \mathrm {M_{validation}} = 5\%$ of $N_t \mathrm {M_{train}}$ (randomly selected, see Supplementary sec. 5.3 for more detail), and test set $\mathrm {M_{test}} = 19$ resulting in $N_t \mathrm {M_{test}} = 1900$. In contrast to the two previous examples, there are 20 cases that FOMs do not converge using a default initialization (${\varvec{X}_h}\left( t^{n-1}, \varvec{\mu }^{(i)} \right)$ as an initial guess for ${\varvec{X}_h}\left( t^n, \varvec{\mu }^{(i)}\right)$).

**Figure 4:**
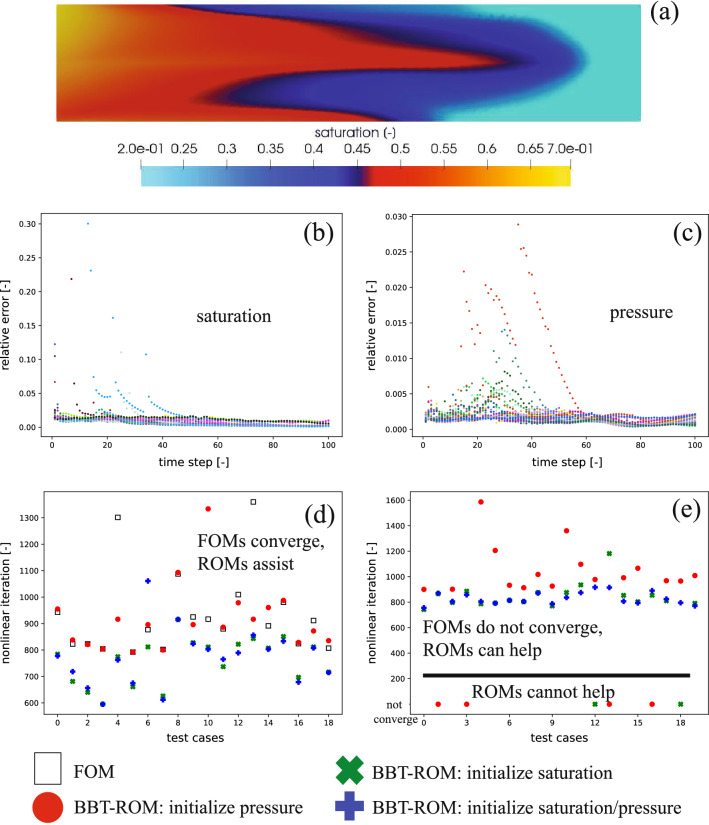
Example 3—results: (**a**) samples of test dataset results of $\varvec{\kappa }_{\mathrm {top}} = 2.42 \times 10^{-10}$ ${\mathrm{m}}^2/{\mathrm{Pa\,s}}$ at 50 days, (**b**) pressure ($p_h$), (**c**) saturation ($s_h$)—relative error (see Eq. 3) of *test dataset* results, (**d**) number of nonlinear iterations for cases that the FOMs converge and we use BBT-ROM to speed up, and (**e**) number of nonlinear iterations for cases that the FOMs do not converge and we use BBT-ROM to improve the convergence. The nonlinear solver iteration using default FOM initialization, ${\varvec{X}_h}\left( t^{n-1}, \varvec{\mu }^{(i)} \right)$ as an initial guess for ${\varvec{X}_h}\left( t^n, \varvec{\mu }^{(i)}\right)$, is shown with a black square, using BBT-ROM pressure initialization is shown with a red dot, using BBT-ROM saturation initialization is illustrated with a green cross, using BBT-ROM saturation and pressure initialization is presented with a blue plus. A number of nonlinear iterations here are an average over all *t* - $0=: t^{0}<t^{1}<\cdots <t^{N} := \tau$ for each $\varvec{\mu }^{(i)}$. The relative error (see Eq. 3) is calculated between FOM and BBT-ROM for each $\varvec{\mu }$ - $\varvec{\kappa }_{\mathrm {top}}$ in this case.

Our results are presented in Fig. 4. We show one of our test dataset where the FOMs converge in Fig. 4a. The relative errors for $s_h$ and $p_h$ are illustrated in Fig. 4b,c, respectively. From these figures, we observe that the relative error of $s_h$ field, is 0.79% (average), 0.09% (minimum), and 30.03 % (maximum). We note that the query—a pair of $t^n, \varvec{\mu }^{(i)}$—which has a relative error that exceeds 5%, is only 0.46% of the total queries ($N_t \mathrm {M_{test}} = 1900$). The relative error of $p_h$ field is much lower than that of the $s_h$ field. The relative error of $p_h$ field, is 0.17% (average), 0.03% (minimum), and 2.89% (maximum).

A number of nonlinear iterations (for parameters for which the FOMs converge) are presented in Fig. 4d. We note that a number of nonlinear iterations here are an average over all *t* - $0=: t^{0}<t^{1}<\cdots <t^{N} := \tau$ for each $\varvec{\mu }^{(i)}$. The default initialization, ${\varvec{X}_h}\left( t^{n-1}, \varvec{\mu }^{(i)} \right)$ as an initial guess for ${\varvec{X}_h}\left( t^n, \varvec{\mu }^{(i)}\right)$, takes 934.68 iterations on average. Using BBT-ROM to initialize $p_h$, the average number of nonlinear iterations is 916.41. With $\widehat{s}_h$ initialization, the average number of nonlinear iterations is 758.57. Using both $\widehat{s}_h$ and $\widehat{p}_h$ initialization, a number of nonlinear iterations are 770.91 on average. These results imply that using BBT-ROM-assisted achieves speed up by 1.95%, 18.84%, or 17.52% for $\widehat{p}_h$ initialization, $\widehat{s}_h$ initialization, or both $\widehat{s}_h$ and $\widehat{p}_h$ initialization, respectively. In terms of wall time, each nonlinear iteration takes 0.2 s using AMD EPYC 7452. As a result, using $\widehat{p}_h$ initialization $\widehat{p}_h$ initialization, $\widehat{s}_h$ initialization, or both $\widehat{s}_h$ and $\widehat{p}_h$ initialization saves 3.65, 35.22, or 32.75 s, respectively. The prediction of BBT-ROM takes about 0.001 s per inquiry, which means 0.01 s for 100 timestamps. This cost is much cheaper compared to the FOM solver itself.

We also present one additional benefit of using BBT-ROM-assisted, namely, cases that diverged using default initialization now converge—see Fig. 4e. Using BBT-ROM to initialize $p_h$ sees 16 out of 20 cases (80%) converging that initially diverged with default initialization. The average number of nonlinear iterations is 1051.23. Using BBT-ROM to initialize $s_h$ sees 18 out of 20 of those cases (90%) now converging, and the average number of nonlinear iterations is 846.57. Using BBT-ROM to initialize $s_h$ and $p_h$ sees all the cases (100%) converging with an average number of nonlinear iterations of 829.39. From these results, by using BBT-ROM-assisted, one can reduce computational cost as well as improve convergence. Again, each nonlinear iteration takes 0.2 s calculating by AMD EPYC 7452. The BBT-ROM training time take approximately 45 min computed using NVIDIA Quadro RTX 5000 Mobile Max-Q.

### Example 4: Phase-field approach for fracture propagation

We utilize a time-dependent phase-field modeling to capture fracture propagation for homogeneous material. The details of the problem statement and the governing equation can be found in Supplementary sec. 4. In the computational domain as shown in Supplementary fig. 7a, the phase-field fracture initiates from the center of the domain and propagates to the left end of the boundary. The fracture propagation is due to the quasi-static tension force boundary condition from the top of the boundary. The fracture propagation speed and the initiation of the fracture time depend on the given tension force. In this example, we have two state variables, displacement ($\varvec{u}_h$) and phase field ($pf_h$). Each has a degree of freedom of 11,646 and 5823, respectively. Our $\varvec{\mu }$ is force exert to the domain ($\mathrm {F}$) and takes values in a certain range $\varvec{\mu } = [t, \mathrm {F}]\in (0.00005, 0.025) \times (0.1, 2.0)]$. We here use only BBT-ROM (Supplementary sec. 5.3) because $\varvec{\mu }$ is homogeneous, similar to Examples 2 and 3, and we have shown its performance in Example 2. Again, we use $\mathrm {N_{en}}=10$ and sub-sample of a quarter of the total training data in this example.

Here, $\mathrm {M_{train}} = 9$ resulting in $N_t \mathrm {M_{train}} = 4509$. To elaborate, we have a training set of $\mathrm {M_{train}}$ generated by choosing 9 values of parameters and, for each parameter set, we collect solutions for 501 timestamps. We select a validation set $N_t \mathrm {M_{validation}} = 5\%$ of $N_t \mathrm {M_{train}}$ (randomly selected, see Supplementary sec. 5.3 for more detail), and test set $\mathrm {M_{test}} = 2$ resulting in $N_t \mathrm {M_{test}} = 1002$. We note that we use only $\widehat{pf}_h$ as an initial guess of $pf_h$ and leave $\varvec{u}_h$ to a default initialization. This shows another advantage of non-intrusive ROMs for coupled multiphysical processes in which we can selectively focus only on the quantities of interest ($pf_h$ in this case or ${\varvec{X}_h}$ in a general sense) without necessarily carrying on the construction of the ROM for $\varvec{u}_h$. We note here that, similar to all previous examples, the default initialization represents cases where we use ${\varvec{X}_h}\left( t^{n-1}, \varvec{\mu }^{(i)} \right)$ as an initial guess for ${\varvec{X}_h}\left( t^n, \varvec{\mu }^{(i)}\right)$. The ROM (BBT-ROM in this case) assists FOM by using $\widehat{\varvec{X}_h}\left( t^n, \varvec{\mu }^{(i)}\right)$ as an initial guess for ${\varvec{X}_h}\left( t^n, \varvec{\mu }^{(i)}\right)$.

**Figure 5:**
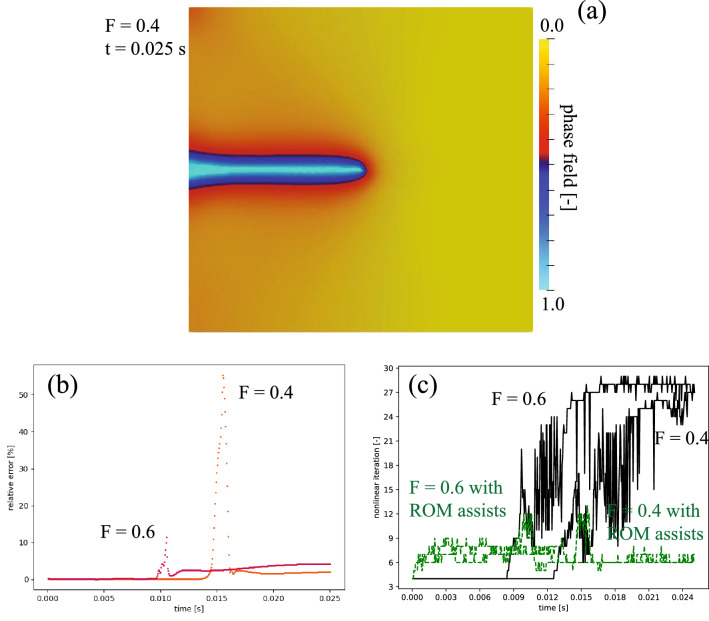
Example 4—results: (**a**) samples of test dataset results of $\mathrm {F} = 0.4$ N at 0.025 s, (**b**) relative error (see Eq. 3) of test dataset results, and (**c**) number of nonlinear iterations for test cases as a function of time with and without ROM assist. Again, the nonlinear solver iteration using default FOM initialization, ${\varvec{X}_h}\left( t^{n-1}, \varvec{\mu }^{(i)} \right)$ as an initial guess for ${\varvec{X}_h}\left( t^n, \varvec{\mu }^{(i)}\right)$, is shown with a black solid line and using BBT-ROM pressure initialization is shown with a green dashed line. The relative error (see Eq. 3) is calculated between FOM and BBT-ROM for each $\varvec{\mu }$—$\mathrm {F}$ as a function of time.

Example 4’s results are presented in Fig. 5. One of the test dataset is shown in Fig. 5a, $\mathrm {F} = 0.4$ at $t=0.025$ s. The relative error (see Eq. 3) for $\widehat{pf}_h$ as a function of time is shown in Fig. 5b. When the crack is propagated, the relative errors are substantially higher, as high as 60%, than those observed in Examples 2 and 3. In contrast, the errors are significantly low (less than 5%) before and after the fracture grows. The nonlinear iterations for two test cases as a function of time are shown in Fig. 5c. We observe that from the beginning of the simulation to right before the fracture propagates, using ROM to assist FOM’s solver has a negative effect as a number of nonlinear iterations increase. However, as the fracture starts to propagate, ROM could assist in reducing the nonlinear iterations significantly. Toward the late stage of the simulation, this assistance becomes even more pronounced as the nonlinear iterations through default initialization grow larger while using ROM-assisted remains almost similar to the trend before the fracture starts to propagate.

The cumulative nonlinear iterations for $\mathrm {F} = 0.4$ are 5615 and 3505 for default initialization and ROM-assisted, respectively. This results in a speed up of 38%. For $\mathrm {F} = 0.6$, the cumulative nonlinear iterations are 8357 and 3319 for default initialization and ROM-assisted, respectively, resulting in a speed up of 61%. These results illustrate that even though the ROM fails to mimic the phase-field modeling for fracture propagation (the relative error is as high as 60%), using ROM-assisted could still reduce the computational cost as much as 61%. In terms of wall time, each nonlinear iteration takes approximately 0.2 s using a single Intel(R) Xeon(R) CPU E5-2680 v3@2.50 GHz. As a result, using $\widehat{pf}_h$ initialization saves approximately 420 s in the nonlinear iterations. The prediction of BBT-ROM takes about 0.001 s per inquiry, which means 0.501 s for 501 timestamps. This cost is much cheaper compared to the FOM solver itself. The BBT-ROM training time takes approximately 30 min using NVIDIA Quadro RTX 5000 Mobile Max-Q.

One additional point for this example is that most of the computational resources are allocated to states before and after fracture propagates for this type of problem. However, what we actually are interested in is (1) when the fracture starts to propagate and (2) when it ends. Hence, we believe that, for the future study, one can build ROM to approximate the fracture dynamics period (start and stop) and simply neglect the building up of energy (before the fracture propagates) period. This way, we can even further save our computational resources.

## Discussion

Even though ROM can deliver a reasonable accuracy at a much lower computational cost, it might not be suitable for an application where precision is paramount. In this case, FOM or a high-fidelity model is still preferable. FOM, however, requires a substantial amount of computational resources, especially in a nonlinear system. Besides, it is not trivial to solve this nonlinear system since, with a non-optimal initial guess or solving algorithm, your solver might not converge at all. Hence, this work proposes a novel approach to achieve the accuracy of FOM performance and improve the convergence of FOM solutions with computational efficiency.

We have illustrated the use of a low-fidelity model (or ROM) as an initial guess (ROM-assisted) to a FOM’s nonlinear solver, which can achieve speed up from 18 to 73% (cGAN-ROM for Example 1 and BBT-ROM for the rest), see Table 1. Moreover, this proposed procedure achieves convergence in all cases that diverge because of the default initial guess (see Example 3), which is a substantial benefit. Since our ROMs are data-driven or non-intrusive, the proposed procedure can easily lend themselves to any nonlinear physics-based problems. We have also illustrated that this procedure is applicable to discretizations based on finite volume (Example 1), finite element (Examples 2 and 4), and hybrid finite volume—finite element (Example 3).

We have summarized the normalized wall time magnitude, normalizing each wall time used by the highest wall time (i.e., FOM with default initialization) and the relative error of ROM in Table 3. Here, we present a relative magnitude of wall time spent for each approach; (1) FOM with default initialization, (2) FOM with ROM assists, and (3) ROM. Since using FOM with default initialization takes the highest wall time, it is represented by $O(10^{0})$. For all tested cases with different ROM approaches, ROM-assisted FOM’s nonlinear solver can save the wall time by one order of magnitude. We note that the prediction time of ROM is insignificant at a scale of $O(10^{-4} \sim 10^{-5})$ compared to FOM’s nonlinear solver. We also show that, even though ROM delivers decent accuracy for Examples 1 and 3 with average relative errors less than 1%, it cannot provide a proper prediction for Examples 2 and 4 (i.e., Example 2 has an average relative errors more than 5%, and Example 4 has the largest relative error of 60%). Hence, using ROM-assisted is essential to obtain a solution which accurate within the tolerance prescribed by the stopping criterion of the nonlinear solver with a much cheaper cost.

**Table 3:** Summary of normalized wall time magnitude and relative error of ROM.

Example		1	2.1	2.2	2.3	3	4	remark
Normalized wall time	FOM	$O(10^{0})$	$O(10^{0})$	$O(10^{0})$	$O(10^{0})$	$O(10^{0})$	$O(10^{0})$	Default initialization
	FOM with ROM assists	$O(10^{-1})$ + $O(10^{-4})$	$O(10^{-1})$ + $O(10^{-5})$	$O(10^{-1})$ + $O(10^{-5})$	$O(10^{-1})$ + $O(10^{-6})$	$O(10^{-1})$ + $O(10^{-5})$	$O(10^{-1})$ + $O(10^{-5})$	$\approx O(10^{-1})$
	ROM	$O(10^{-4})$	$O(10^{-5})$	$O(10^{-5})$	$O(10^{-6})$	$O(10^{-5})$	$O(10^{-5})$	cGAN-ROM for Ex. 1 BBT-ROM for Ex. 2, 3
Average relative error of ROM compared to FOM		0.13%	10.35%	6.17 %	17.17%	0.17%, 0.79%	2.33%	

ROM uses much less computational time (at least four orders of magnitude less) than the FOM nonlinear solver; hence, using ROM to assist FOM reduces a computational cost by one order of magnitude. Normalized wall time is calculated by normalizing each wall time used by the highest wall time (i.e., FOM with default initialization). For Example 3, there are two primary variables, $p_h$ and $s_h$; therefore, we report two relative error values for these two variables, respectively.

Another benefit of using ROM-assisted is it can improve the convergence rate. As a system of nonlinear PDEs is not straightforward to solve, and the convergence rate highly depends on the initial guess, preconditioner, or solving algorithm^32–35^, we have shown that ROM-assisted can converge all diverged cases (with default initialization). This characteristic is preferable and beneficial to many engineering applications.

However, there are computational costs associated with the training of these non-intrusive ROMs. The majority of costs is allocated to the generation of the training data itself. We speculate that an adaptive sampling technique^68–70^ or incorporating physical information^71,72^ could reduce the required number of training data while maintaining similar accuracy. The cost of training of BT-ROM and BBT-ROM ranges from 30 to 45 min using a graphic processing unit (GPU), while the training of cGAN-ROM uses a significantly higher cost of 4 h through a GPU (see Table 1). We note that without GPU computing, the training time of these models is impractical, which might hinder the applicability of these models. It should be noted that our ROMs are trained on a specific topology (i.e., fixed degrees of freedom, coordinates, and connectivity) for each problem. As a result, if we alter the topology, we need to retrain our ROMs.

## Conclusion

Through this work, we have illustrated that by using reduced order modeling (ROM) as an initial guess to a nonlinear solver of full order modeling (FOM), we can reduce computation cost (fewer nonlinear iterations) and improve the convergence rate. To elaborate on these benefits, we showcase our framework through four different physics problems discretized by different numerical methods (e.g., finite volume or finite element). Our results show that our approach speeds up nonlinear solvers by 18–73%. Besides, our framework improves the convergence of FOMs that would have otherwise diverged with default initial guesses. We want to emphasize that as our ROMs are data-driven and non-intrusive, we can apply them to any nonlinear physics-based problem.

## Supplementary Information

Supplementary Information.

## Data Availability

Our ROMs and all data generated or analyzed during this study will be available publicly through the Sandia National Laboratories software portal—a hub for GitHub-hosted open source projects (https://github.com/sandialabs) with Sandia National lab’s internal review and approval process.
