# Supplementary material for: Enhancing high-fidelity nonlinear solver with reduced order model
Source: Sci Rep. 2022 Nov 23;12:20229. doi: 10.1038/s41598-022-22407-6 (PMC9684583; doi:10.1038/s41598-022-22407-6)
Supplement: Supplementary file 1 — Supplementary Information. [file 41598_2022_22407_MOESM1_ESM.pdf]

Supplementary information

Supplementary sec. 1 Richards' equation with heterogeneous material

We first describe the Richards' equation, which is used to mimic the flow of water through unsaturated porous media[1]. Throughout this manuscript, we focus on using heterogeneous conductivity fields (κ) as a parameter $\boldsymbol{\mu}$, meaning we solve a state variable, pressure head h_p, of Richards' equation with given a range of κ values.

The steady-state Richards' equation is described by

$$\nabla \cdot k_r(s_p)\kappa \nabla h_p = q, \qquad \text{(Supplementary eq. 1)}$$

where $k_r(s_p)$ is a relative permeability as a function of suction pressure s_p. The Van Genuchten-Mualem relative permeability model is used as follows

$$k_r(s_p) = \frac{\left\{1 - |\alpha s_p|^{N_{sp}-1}\left[1+|\alpha s_p|^{N_{sp}}\right]^{-m_{sp}}\right\}^2}{\left[1+|\alpha s_p|^{N_{sp}}\right]^{m_{sp}/2}}. \qquad \text{(Supplementary eq. 2)}$$

Here, α and N_{s_p} are constant depending on soil types set as 0.375 and 1.25, respectively, m_{s_p} is calculated by $m_{s_p} = 1 - \frac{1}{N_{s_p}}$. Again, κ represents a heterogeneous (intrinsic) conductivity, h_p is a hydraulic head, and q is a sink/source term. We solve the Richards' equation in a dimensionless setting, using finite volume discretization - see `https://github.com/OrchardLANL/DPFEHM.jl` for the detailed implementations. The problem domain and boundaries are described in Supplementary fig. 1. The computational domain $\Omega \subset \mathbb{R}^2$ is defined as $\Omega = (0,100) \times (0,10)$, i.e., $l_1 = 100$ and $l_2 = 10$. In this example, the parameters $\boldsymbol{\mu}$ are the heterogeneous conductivity fields κ with mean $\log(\kappa)$ of 0.0, the $\log(\kappa)$ standard deviation of 0.25, and the correlation length is 10. We employ a finite volume discretization where the pressure is h_p is discretized and the saturation is given by

$$s_p = \begin{cases} 1 & h_p \geq 0 \\ (1+|\alpha h_p|^{N_{sp}})^{-m_{sp}} & h_p < 0 \end{cases} \qquad \text{(Supplementary eq. 3)}$$

The nonlinear discretized equations are solved using the "nlsolve" function from `https://github.com/JuliaNLSolvers/NLsolve.jl` with the default settings, which uses a Newton iteration with a trust region. The boundary conditions are as follows, on $\partial\Omega_1$ and $\partial\Omega_3$, there are zero flux boundaries, on $\partial\Omega_2$, there is a fixed pressure boundary with $h_p = 0$, corresponding to a saturated water table, and on $\partial\Omega_4$, there is a fluid source with $q = 10^{-2}$. Note that we leave κ and q dimensionless, but any consistent dimensionalization could be used.

Supplementary sec. 2 Contact problems with hyperelasticity material

The second physical problem we focus on is contact problems in the large deformation context, which is applicable to many engineering applications such as biomedical engineering, material science, and the aerospace industry[2–4]. This work is an extension of the Hertzian contact problem in the resource 'Numerical Tours of Computational Mechanics with FEniCS'[5]. For this problem, the rigid indenter with a spherical surface can be approximated by a parabolic equation instead of explicitly modeled. As long as the indenter radius, $\mathrm{In_R}$ is sufficiently large with respect to the contact region size, $\mathrm{In_D}$, the spherical surface to be approximated by a parabolic function,

$$\mathrm{In}(x,y) = -\mathrm{In_D} + \frac{1}{2\mathrm{In_R}}(x^2+y^2) \quad \text{about origin} \qquad \text{(Supplementary eq. 4)}$$

where we have defined the plane of indentation to be the $x-y$ plane. The normal component of the gap function defines the gap between the indenter, $\mathrm{In}(x,y)$, and the normal displacement of the displacement vector, u_N

$$g_N = \mathrm{In}(x,y) - u_N. \qquad \text{(Supplementary eq. 5)}$$

where we assume only normal contact and no tangential contact contributions (e.g. from friction).

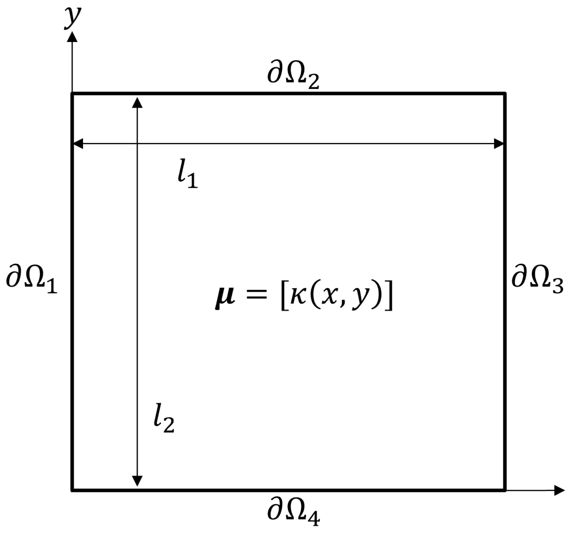

Supplementary fig. 1. Richards' equation with heterogeneous material: domain, its boundaries, and parameter space.

The Karush Kuhn Tucker (KKT) optimization equations mathematically define the cases of contact possible

$$\begin{aligned} g_N &\geq 0 \\ \lambda_N &\geq 0 \text{ on } \partial\Omega_c \\ \lambda_N g_N &= 0. \end{aligned} \qquad \text{(Supplementary eq. 6)}$$

where $\partial\Omega_c$ represents the contact surfaces and λ_N is a lagrange multiplier, equivalent to the contact pressure. The definition of the MacKauley bracket is useful for defining regions between no contact and contact given by

$$\langle X \rangle = \frac{X + |X|}{2}. \qquad \text{(Supplementary eq. 7)}$$

Here, X could represent any variables, such as the normal component of the gap function, g_N, in this case. The governing equation is the mechanical equilibrium equation in the reference configuration, where the domain before deformation is represented with Ω_0 and after deformation with Ω_i where i represents different states of deformation).

$$\begin{aligned} \nabla_X \cdot \mathbf{P} + \mathbf{B} = 0 &\quad \text{in } \Omega_0 \\ \mathbf{u} = \bar{\mathbf{u}} &\quad \text{on } \partial\Omega_D \\ \mathbf{P} \cdot \mathbf{N} = \overline{\mathbf{T}} &\quad \text{on } \partial\Omega_N \end{aligned} \qquad \text{(Supplementary eq. 8)}$$

The 1st Piola Kirchhoff stress tensor, **P**, is a function of Young's modulus (E) and Poisson ratio (ν). The normal vector to the domain boundary, $\partial\Omega$ is defined as **n**. The body and traction forces are **B** and **T**, respectively. The primary variable in the system is the displacement vector, **u**. The over-bar indicates quantities prescribed, where displacement is prescribed on the boundary $\partial\Omega_D$ where 'D' indicated the Dirichlet boundary condition, and traction is prescribed on $\partial\Omega_N$ where 'N' is the Neumann boundary condition. We note that $\partial\Omega_c$ where the contact is applied is a subset of the Dirichlet domain, $\partial\Omega_D$.

To obtain the weak form, we multiply the mechanical equilibrium equation with a test function, integrate over the domain Ω_0, conduct integration by parts, and recognize the traction term

$$\int_{\Omega_0} \mathbf{P} : \nabla_X(\delta \mathbf{u})\, dV = \int_{\Omega_0} \mathbf{B} \cdot \delta \mathbf{u}\, dV + \int_{\partial\Omega_N} \mathbf{T} \cdot \delta \mathbf{u}\, dS. \qquad \text{(Supplementary eq. 9)}$$

We utilize the penalty method of enforcing contact, which considers the addition of the following penalty term to the overall energy, Π. Specifically, we consider cases of only normal contact, otherwise known as pure stick considerations read

$$\Pi_N^P = \frac{1}{2}\int_{\partial\Omega_c} \mathrm{k_{pen}}(g_N)^2 dA \quad \text{where } \mathrm{k_{pen}} > 0 \qquad \text{(Supplementary eq. 10)}$$

Taking the variation of Supplementary eq. 10, the addition to the weak form is as follows

$$C_N^P = \mathrm{k_{pen}} \int_{\partial\Omega_c} \langle -g_N \rangle \delta u_N dA. \qquad \text{(Supplementary eq. 11)}$$

Therefore, the full weak form which holds for finite deformations is

$$\mathrm{k_{pen}} \int_{\partial\Omega_c} \langle -g_N \rangle \delta u_N \, dS + \int_{\Omega_0} \mathbf{P} : \nabla(\boldsymbol{\delta}\mathbf{u})\, dV - \int_{\Omega_0} \mathbf{B}\cdot\boldsymbol{\delta}\mathbf{u}\, dV - \int_{\partial\Omega_N} \mathbf{T}\cdot\boldsymbol{\delta}\mathbf{u}\, dS = 0 \qquad \text{(Supplementary eq. 12)}$$

where the MacKauley bracket enforces regions of contact and no contact. To approximate $\mathbf{u}$ (i.e., $\mathbf{u}_h$), we use a continuous Galerkin approximation of the first order. We use PETSc SNES as a nonlinear solver and MUMPS as a linear solver[6] with absolute and relative tolerances of 1×10^{-6} and 1×10^{-16}, respectively. We utilize a backtracking line search with slope descent parameter of 1×10^{-4}, initial step length of 1.0, and quadratic order of the approximation. We have three test scenarios corresponding to using (1) Poisson's ratio and indentation depth as parameters - $\boldsymbol{\mu} = [\nu, \mathrm{In_D}]$ (see Supplementary sec. 2.1), (2) indentation radius and indentation depth as parameters - $\boldsymbol{\mu} = [\mathrm{In_R}, \mathrm{In_D}]$ (see Supplementary sec. 2.2), and (3) indentation location is a parameter - $\boldsymbol{\mu} = [x, y]$ (see Supplementary sec. 2.3). Body and traction forces, $\mathbf{B}$ and $\mathbf{T}$, are neglected in all cases.

Supplementary sec. 2.1 Young's modulus and indentation depth are parameters

The first test scenario uses Poisson's ratio and indentation depth as parameters - $\boldsymbol{\mu} = [\nu, \mathrm{In_D}] = (0.1, 0.4) \times (0.1, 0.3)$. Its domain and boundaries are shown in Supplementary fig. 2. The top surface is a contact surface $\partial\Omega_c$ with an indentation point at the corner of the domain - $(0.0, 0.0, 1.0)$. We apply roller boundary condition to all other surfaces. We set $l_1 = 1.0$ m, $l_2 = 1.0$ m, and $l_3 = 1.0$ m. The degree of freedom for this FOM - $\mathbf{u}_h$, an unstructured mesh, is 3993, which means 1331 degrees of freedom for each displacement in x-, y-, and z-directions. We set $\mathrm{In_R} = 0.5$ m, $\mathrm{k_{pen}} = 1 \times 10^4$, and E = 10 Pa.

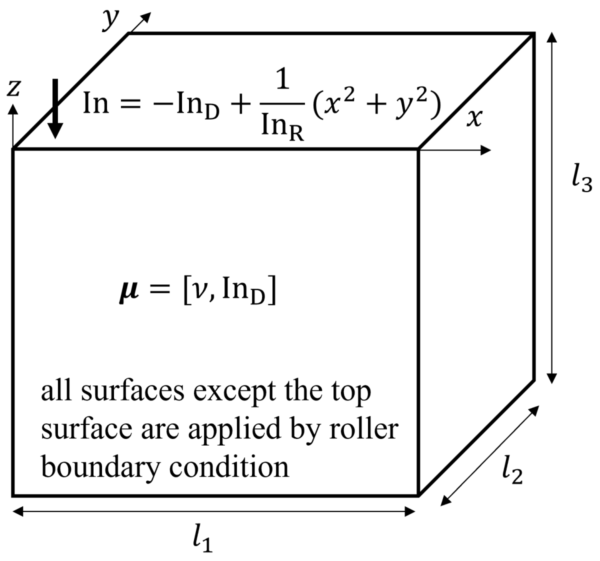

Supplementary fig. 2. Contact problem - Young's modulus and indentation depth are parameters: domain, its boundaries, and parameter space.

Supplementary sec. 2.2 Indentation radius and indentation depth are parameters

The second scenario uses indentation radius and indentation depth as parameters - $\boldsymbol{\mu} = [\mathrm{In_R}, \mathrm{In_D}] = (0.15, 0.4) \times (0.1, 0.4)$. The domain and boundaries are shown in Supplementary fig. 3. Again, the top surface is a surface where contact will occur in the area of $\partial\Omega_c$, with an indentation point at the corner of the domain - $(0.0, 0.0, 1.0)$, and we apply roller boundary condition

to all other surfaces. We set $l_1 = 1.0$ m, $l_2 = 1.0$ m, and $l_3 = 1.0$ m. Similar to the previous scenario, the degree of freedom for this FOM - $\mathbf{u}_h$, an unstructured mesh, is 3993, which means 1331 degrees of freedom for each displacement in x-, y-, and z-directions. We set $\nu = 0.3$, $\mathrm{k_{pen}} = 1 \times 10^4$, and $\mathrm{E} = 10$ Pa.

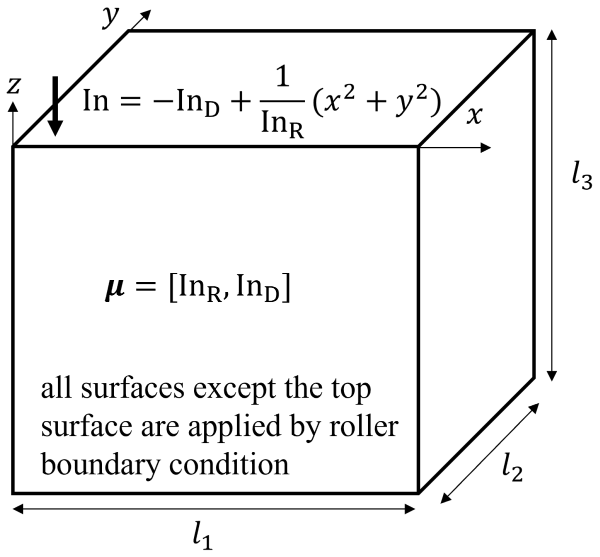

Supplementary fig. 3. Contact problem - Indentation radius and indentation depth are parameters: domain, its boundaries, and parameter space.

Supplementary sec. 2.3 Indentation location is a parameter

The third scenario uses indentation location as parameters - $\boldsymbol{\mu} = [x, y] = (-0.3, 0.3) \times (-0.3, 0.3)$. To elaborate, we randomly push the domain according to the values of x and y at the contact surface, which is a subset of top surface and is only defined as where there will be contact according to the KKT condition. Again, we apply roller boundary condition to all other surfaces. To prevent the boundary effects, we limit values of x and y within the black square shown in Supplementary fig. 4. We set $l_1 = 3.0$ m, $l_2 = 3.0$ m, $l_3 = 0.4$ m, $l_4 = 2.5$ m, and $l_5 = 2.5$ m. The degree of freedom for this FOM ($\mathbf{u}_h$) is 70602, an unstructured mesh, which means 23534 for each displacement in x-, y-, and z-directions. We set $\mathrm{In_R} = 0.5$ m, $\nu = 0.3$, $\mathrm{k_{pen}} = 1 \times 10^4$, and $\mathrm{E} = 10$ Pa.

Supplementary sec. 3 Two-phase flow in layered porous material

Here we consider incompressible multiphase flow with gravity and capillary pressure. The conservation equation for phase α can be written as

$$\phi \frac{\partial S_\alpha}{\partial t} + \nabla \cdot \mathbf{q}_\alpha = 0, \qquad \text{(Supplementary eq. 13)}$$

where t is time, ϕ porosity, $\mathbf{q}_\alpha$ is the Darcy velocity, and S_α is the saturation.

Darcy's law for phase α is read

$$\mathbf{q}_\alpha = \frac{k_{r\alpha} \boldsymbol{\kappa}}{\mu_\alpha}(-\nabla p_\alpha + \rho_\alpha \mathbf{g}), \qquad \text{(Supplementary eq. 14)}$$

where ρ_α is the density, $\mathbf{g}$ is the gravity vector and p_α is the pressure. $k_{r\alpha}$, $\boldsymbol{\kappa}$, and μ_α are the relative permeability, permeability tensor, and viscosity, respectively.

Considering a wetting and non-wetting phase and including capillary pressure

$$p_c = p_{nw} - p_w, \qquad \text{(Supplementary eq. 15)}$$

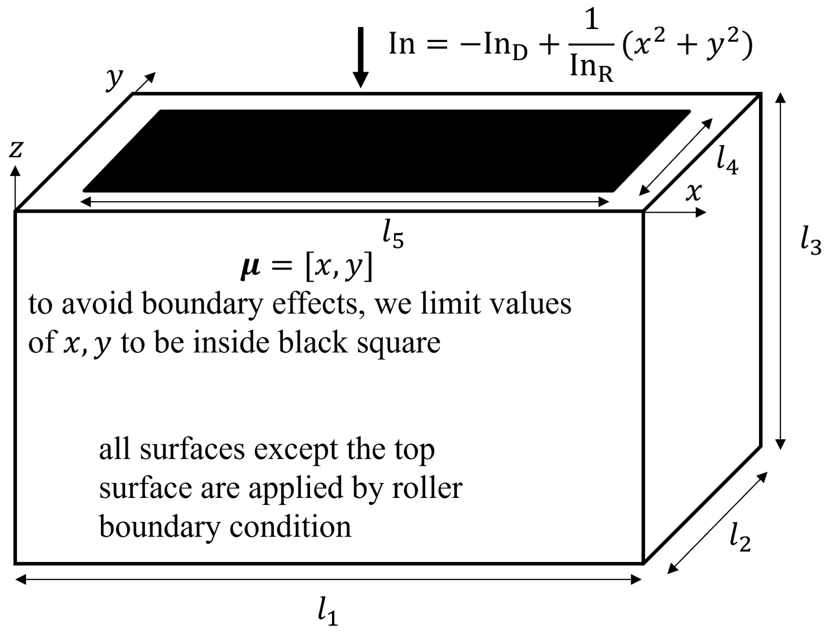

Supplementary fig. 4. Contact problem - Indentation location is a parameter: domain, its boundaries, and parameter space.

where p_w and p_{nw} are the pressures of the wetting and non-wetting phases, respectively. p_c is the capillary pressure.

Finally, the saturation constraint is given by

$$S_w + S_{nw} = 1, \qquad \text{(Supplementary eq. 16)}$$

where S_w and S_{nw} are the saturations of the wetting and non-wetting phases, respectively.

The Imperial College Finite Element Reservoir SimulaTor (IC-FERST) is used here for the solutions of the reported equations[7–10]. High order methods are used whenever possible both in time (θ-method based on total variation diminishing (TVD) criterion[11]) and space (Double Control Volume Finite Element method, described in[10], using a normalized variable diagram (NVD) scheme following the upwind direction).

Supplementary sec. 3.1 Nonlinear solver

To solve the discretized form of the nonlinear system of equations formed by Eqs. (Supplementary eq. 13), (Supplementary eq. 14), (Supplementary eq. 15) and (Supplementary eq. 16) a Picard iterative method is used[9]. Supplementary fig. 5 describes the overall method. The solver comprises three main loops, where the solid line represents the time loop, the dotted line denotes the coupling between the saturation and pressure (nonlinear outer loop), and the dashed line iterates over saturation and velocity (nonlinear inner loop). In the latter loop, after calculating the saturation, the velocity is updated. Using the new velocity, a new saturation is estimated, and the process continues until the maximum iterations or convergence are reached. Following that, new pressure is estimated in the outer loop based on the new saturation, and the velocity is calculated from the pressure estimation. Then, a new inner loop iteration starts and the process repeats until convergence, or the maximum number of iterations is reached. Within the nonlinear loops, the linear system of equations is solved using GMRES and multigrid as preconditioner[6].

Supplementary sec. 3.2 Problem description

The parameters in this example are set as - $\boldsymbol{\mu} = [t, \boldsymbol{\kappa}_{\text{top}}] = [(0.0, 100.0), (1.08 \times 10^{-11}, 9.97 \times 10^{-10})]$. Our model treats the time domain as one of the parameterized variables[12, 13]. Its domain and boundaries are shown in Supplementary fig. 6. We set $l_1 = 100$ m, $l_2 = 10$ m, and $l_3 = 10$ m. In this example, we have two state variables, pressure (p_h) and saturation (s_h), and both variables have the same number of degrees of freedom of 2548. We set the permeability of the bottom layer to 1.0×10^{-13} m^2 and the porosity in the top and bottom layers to 0.1 and 0.2, respectively. The vertical permeability is equal to 10% of the horizontal permeability. We inject one phase on the left and produce both phases on the right by imposing a fixed pressure on both boundaries. The top and bottom boundaries are closed to flow. The viscosity and density of the injected and displaced phases are (1.0×10^{-3} Pa$\cdot$s, 1.0×10^3 kg/m^3) and (5.0×10^{-3} Pa$\cdot$s, 7.0×10^2 kg/m^3), respectively. It is worth mentioning that for defining $\boldsymbol{\mu}$ in this example, we use the permeability divided by the viscosity of the injected phase. A Brooks–Corey

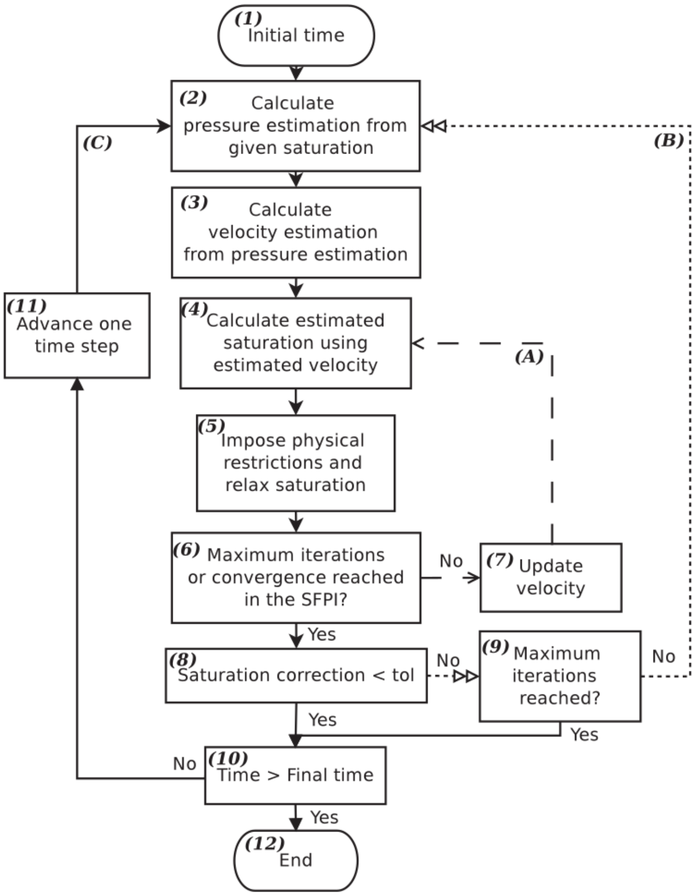

Supplementary fig. 5. Flow chart of the nonlinear solver for the two-phase flow in porous media. Reprinted from Salinas et al.[9].

model[14] is used for the relative permeability and capillary pressure. The rock-fluid properties are the same as test cases 1 and 2 in Silva et al.[15].

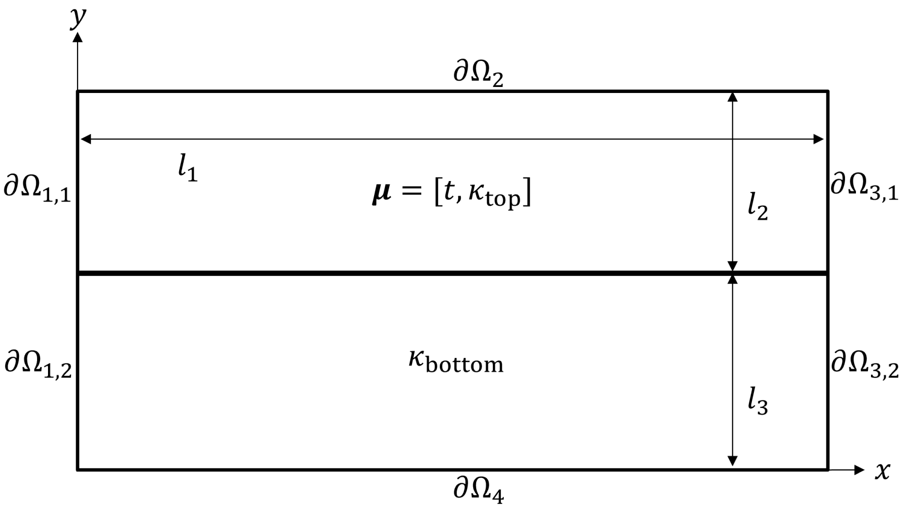

Supplementary fig. 6. Two-phase flow in layered porous material: domain, its boundaries, and parameter space.

Supplementary sec. 4 Phase-field approach for fracture propagation

In the computational domain $\Omega(t) = [0,1]^2 \times (0, 0.025]$, the following energy minimization problem is considered for fracture propagation,

$$E_\varepsilon(\boldsymbol{u}, pf) = \frac{1}{2}\int_\Omega \left((1-\kappa)pf^2 + \kappa\right)\sigma(\boldsymbol{u})dx + G_c \int_\Omega \left(\frac{(1-pf)^2}{2\varepsilon} + \frac{\varepsilon}{2}|\nabla pf|^2\right) dx, \qquad \text{(Supplementary eq. 17)}$$

where $\kappa \ll 1$ is a numerical regularization term, $\boldsymbol{u} : \Omega \to \mathbb{R}^2$ is the vector displacement function and $pf : \Omega \to [0,1]$ is the scalar phase-field function. Here, the $pf = 0$ defines the fractured zone and $pf = 1$ indicates the non-fractured zone, where $\varepsilon > 0$ is a regularization parameter which is the critical length of the diffusive zone with $pf \in (0,1)$. Thus, only the fracture energy is considered if $pf = 0$ since the bulk energy (non-fractured zone) vanishes ($\kappa \approx 0$). On the other hand, only the bulk energy is considered if $pf = 1$ since the fracture energy is zero ($1 - pf = \nabla pf = 0$). Both nonzero bulk and fracture energy will be interpolated in the diffusive zone ($pf \in (0,1)$). Moreover, the energy functional equation will be minimized with the irreversibility condition $\partial_t pf \leq 0$. This condition only allows the fracture to propagate, pf decreases from 1 to 0 in time, and the propagation is implemented in terms of quasi-static assumptions. Here, we consider classical linear elasticity by setting $\sigma(\boldsymbol{u}) = 2\mu e(\boldsymbol{u}) + \lambda tr(e(\boldsymbol{u}))I$, where $e(\boldsymbol{u}) := \frac{1}{2}(\nabla u + \nabla u^T)$ is the symmetric strain tensor, μ and λ are lame material parameters. This energy functional is established in[16,17] based on Griffith's theory[18] and Ambrosio-Tortorelli energy functional[19].

Supplementary sec. 4.1 Numerical methods

We solve the minimization problem

$$\min E_\varepsilon(\boldsymbol{u}, pf) \text{ subject to } \partial_t pf \leq 0$$

where time t enters through the time-dependent boundary conditions on the boundaries of Ω. Here, the weak formulations are derived by employing a monolithically coupled Euler-Lagrange system as in[20,21]. Then, the linear continuous Galerkin finite element method and first order backward Euler method are utilized for spatial and temporal discretizations, respectively. In the numerical algorithm, two Newton iterations are combined. The first Newton iteration considers the nonlinearity in the energy functional and the second Newton iteration is for the primal-dual active set strategy[20] for the irreversibility constraint. To ensure the efficiency, the mesh is pre-refined near the fracture path for this example. The programming software is based on the deal.II[22] and implementation in[20,21]. In this work, the ROM framework will provide an initial data for the combined Newton iterations for each timesteps, i.e., we use $\widehat{\boldsymbol{X}_h}\left(t^n, \boldsymbol{\mu}^{(i)}\right)$ as an initial guess for $\boldsymbol{X}_h\left(t^n, \boldsymbol{\mu}^{(i)}\right)$. While the default initialization uses $\boldsymbol{X}_h\left(t^{n-1}, \boldsymbol{\mu}^{(i)}\right)$ as an initial guess for $\boldsymbol{X}_h\left(t^n, \boldsymbol{\mu}^{(i)}\right)$.

Supplementary sec. 4.2 Problem Description

In this example, we test a simple quasi-static fracture evolution in the domain illustrated in Supplementary fig. 7. Here, the

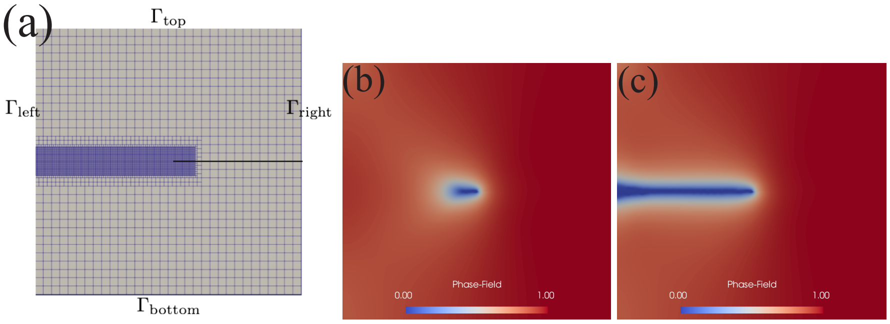

Supplementary fig. 7. (a) setup for the quasi-static propagation of the fracture. (b and c) an example of the propagation of fracture illustrated by the phase-field values.

single edge notched test is implemented with the following boundary conditions: $\boldsymbol{u}_y = 0$ and traction free in x-direction on the Γ_{bottom}, $\boldsymbol{u}_x = 0$ and $\boldsymbol{u}_y = \tilde{u} \times t$ on Γ_{top}, where t is the current time. Traction free boundary conditions for all directions are given on Γ_{left} and on Γ_{right}. For the training and testing, different values for $\tilde{u}$ are chosen. In this case, we set $\tilde{u} = 0.1, 0.25, 0.5, 0.75, 1., 1.25, 1.5, 1.75$, and 2.0 for the training and used $\tilde{u} = 0.2, 0.4, 0.6$ and 1.2 for the testing. The material properties are set as $\mu = 80.77kN/m^2$, $\lambda = 121.15kN/m^2$, and $G_c = 2.7N/m$. The numerical parameters are given as $\kappa = 10^{-10}$, $\varepsilon = 2h$, where $h = 0.0078125$, $\Delta t = 5 \times 10^{-5}$, and the number of time steps are 500. As the time increases, the fracture will propagate from the center to the left end of the boundary. See Supplementary fig. 7 for an example.

Supplementary sec. 5 Description of each reduced order model

Throughout this section, we describe the four different ROMs that are used in the main text - **Results** section. To sum up, the first model utilizes the conditional generative adversarial networks (cGAN) for learning a forward and an inverse solution operator of partial differential equations (PDEs)[23], see Supplementary sec. 5.1. This model takes inputs as heterogeneous fields, which could be material properties, boundary conditions, or masked pixels, and subsequently estimates fields of quantities of interest. Recently, it has been extended to accommodate homogeneous continuous variables (e.g., time domain) as well as a combination among heterogeneous and homogeneous variables[24]. These models operate on a structured mesh.

The rest of the ROM frameworks can take only homogeneous continuous variables. It has been developed to handle highly nonlinear problems with a structured mesh[12] and has subsequently been extended to accommodate both linear and nonlinear problems with an unstructured mesh[13]. This ROM relies on the combination of an autoencoder (AE) and Barlow Twins (BT) self-supervised learning[25], where BT maximizes the information content of the embedding with the latent space through a joint embedding architecture, see[13] and Supplementary sec. 5.2. In this paper, we also extend this model to handle unbalanced datasets (i.e., only a tiny part of a domain is altered while the rest remains unchanged) through the boosting algorithm, see Supplementary sec. 5.3. We here focus on non-intrusive or data-driven ROM; however, we also provide a comparison of these models with an intrusive ROM, see Supplementary sec. 5.4.

Remark 1 *The data compression techniques utilized in Supplementary sec. 5.1, Supplementary sec. 5.2, and Supplementary sec. 5.3 yield nonlinear manifolds while the one employed in Supplementary sec. 5.4 yields linear manifolds. This is because the intrusive ROM or in-ROM recovers the quantities of interest through the Galerkin projection, which relies on linear expansions of compressed manifolds.*

Supplementary sec. 5.1 Reduced order modeling with conditional generative adversarial networks

We provide a brief description of the cGAN-ROM[23,24]. This ROM is a data-driven model order reduction for physics-based problems. It utilized the cGAN concept[26–28] and more specifically image-to-image translation[29,30]. The summary of this framework is provided in Supplementary fig. 8, and the comprehensive details could be found in Kadeethum et al.[23]. In short, the cGAN-ROM begins with an initialization of the training set $\boldsymbol{\mu}$ (i.e., a set of heterogeneous conductivity fields). Subsequently, we query a FOM for each parameter $\boldsymbol{\mu}$ in the training set. We then train our ROM using the heterogeneous conductivity fields as an input and quantities of interest, $\boldsymbol{X}_h$ as an output. We will be discussing the model architecture and training procedures in the following paragraphs. Lastly, during the online phase, given fields of the heterogeneous conductivity, the model will deliver $\widehat{\boldsymbol{X}}_h$, an approximation of $\boldsymbol{X}_h$.

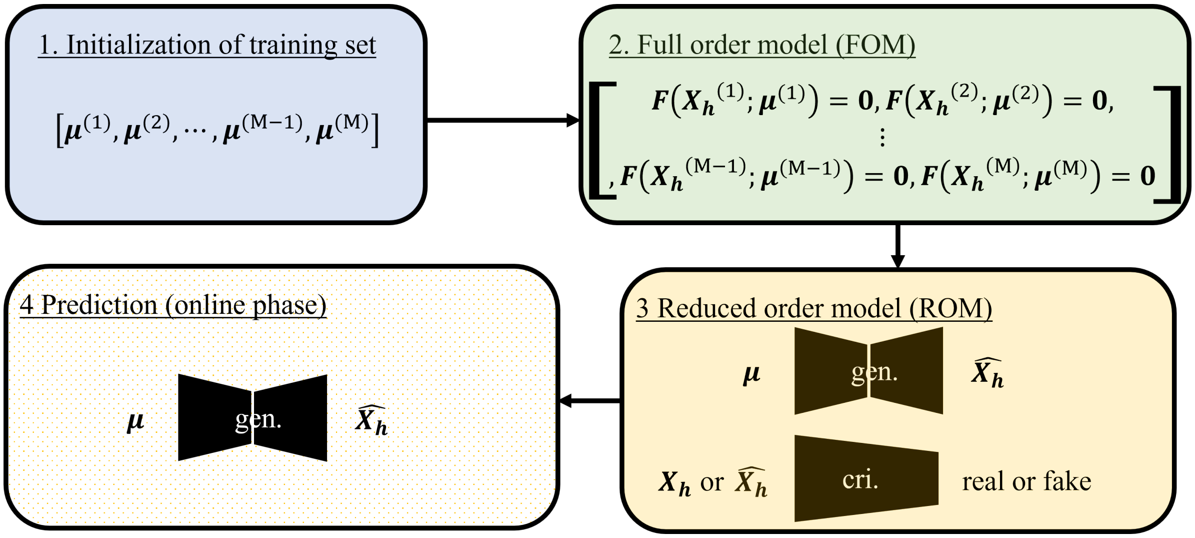

Supplementary fig. 8. Summary of cGAN-ROM - Gen. represents generator, and Cri. is critic. We note that $\boldsymbol{X}_h$ is quantities of interest obtained from FOM, and $\widehat{\boldsymbol{X}}_h$ is an approximation of $\boldsymbol{X}_h$ obtaining from ROM in forward modeling setting. $\boldsymbol{\mu}$ is a set of parameterized spatial fields.

This cGAN-ROM is built upon a combination of U-net[31] as a generator and patch critic (i.e., the critic output is not a single value, but a matrix[32]). The Wasserstein loss or W loss[33,34]

$$\min_{G}\max_{D}\left[\ell_a+\lambda_r\ell_r+\lambda_p\wp_p\right]. \quad \text{(Supplementary eq. 18)}$$

is used as a training metric. Here, $\mathbb{B}$ is batch size and ℓ_a is the Earth mover's distance defined as

$$\ell_a=\frac{1}{\mathbb{B}}\sum_{i=1}^{\mathbb{B}}D(I_i,O_i)-\frac{1}{\mathbb{B}}\sum_{i=1}^{\mathbb{B}}D\left(I_i,\widehat{O}_i\right), \quad \text{(Supplementary eq. 19)}$$

where $D(I_i,O_i)$ is an output matrix from the patch critic for each training sample i using a real output O; $D\left(I_i,\widehat{O}_i\right)$, on the other hand, is an output matrix from the patch critic using an approximated output $\widehat{O}$ for each sample i produced by the generator ($G(I_i)=\widehat{O}_i$). λ_p denotes a gradient penalty constant set to 10 throughout this study, $\wp_p$ gradient penalty regularization, which is used to enforce Lipschitz continuity of weight matrices ($\mathbf{W}$). To enforce the Lipschitz continuity, we use the gradient penalty method[34]. The gradient penalty adds a regularization term ($\lambda_p\wp_p$) to the loss function Supplementary eq. 18. This term penalizes the critic when its gradient norm is higher than one by

$$\wp_p=\frac{1}{\mathbb{B}}\sum_{i=1}^{\mathbb{B}}\left(\|\nabla D(I_i,\bar{O}_i)\|_2-1\right)^2, \quad \text{(Supplementary eq. 20)}$$

where $\bar{O}_i$ is a mixing between $\widehat{O}_i$ and O_i defined by

$$\bar{O}=\varepsilon_i O+(1-\varepsilon_i)\widehat{O}_i. \quad \text{(Supplementary eq. 21)}$$

Here, we randomly select ε_i for each $\bar{O}_i$ from a uniform distribution on the interval of $[0,1)$. The λ_r is a constant set as $\lambda_r=500$ throughout this paper. The ℓ_r is read

$$\ell_r=\frac{1}{\mathbb{B}}\sum_{i=1}^{\mathbb{B}}\left|\widehat{O}_i-O_i\right|. \quad \text{(Supplementary eq. 22)}$$

We follow Kadeethum et al.[23,24] and use the adaptive moment estimation (ADAM) algorithm[35] to train the framework. The learning rate (η) is calculated as[36]

$$\eta_c=\eta_{\min}+\frac{1}{2}(\eta_{\max}-\eta_{\min})\left(1+\cos\left(\frac{\text{step}_\text{c}}{\text{step}_\text{f}}\pi\right)\right) \quad \text{(Supplementary eq. 23)}$$

where η_c is a learning rate at step step_c, $\eta_{\min}$ is the minimum learning rate, which is set as 1×10^{-16}, $\eta_{\max}$ is the maximum or initial learning rate, which is selected as 1×10^{-4}, step_c is the current step, and step_f is the final step. To prevent our networks from overfitting behavior, we follow early stopping and generalized cross-validation criteria[37,38]. Note that instead of literally stopping our training cycle, we only save the set of trained weight and bias to be used in the online phase when the current validation loss is lower than the lowest validation from all the previous training cycle.

Remark 2 *The cGAN-ROM is only applicable to a steady-state solution. The cGAN-ROM has been extended to continuous cGAN-ROM or CcGAN-ROM in Kadeethum et al.*[24]*. Since the problem at hand (Supplementary sec. 1) does not require transient solutions, we here only apply the cGAN-ROM.*

Supplementary sec. 5.2 Reduced order modeling with Barlow Twins self-supervised learning

Proper orthogonal decomposition (POD) is traditionally used as a data compression tool (i.e., linear manifold approach). However, POD-based solutions on a linear manifold are often restrictive for highly nonlinear problems where reduced spaces lie in nonlinear manifolds. Recently, nonlinear compression using autoencoder-based deep learning (DL-ROM)[12,39,40] has been used to construct nonlinear manifolds, and it has shown more promising predictive capabilities than linear manifolds for nonlinear problems. Kadeethum et al.[12] illustrate that there are two essential issues for DL-ROM. First, the nonlinear approach outperforms its linear counterpart in specific settings, but the opposite can occur in other settings. This is because POD provides

the optimal data compression in a linear manifold for the problems with fast-decaying Kolmogorov's n-width[41,42]; hence, the DL-ROM approach could not exceed the level of POD accuracy for problems that naturally lie within linear manifolds. However, the nonlinear manifold approach outperforms the linear manifold method for problems with slowly decaying Kolmogorov's width. Second, although the nonlinear approach excels in very complex nonlinear problems, it relies on convolutional operators, hindering its application for unstructured meshes and limiting DL-ROM approaches to less practical issues.

Kadeethum et al.[13] has proposed a unified data-driven ROM that bridges the performance gap between the linear and nonlinear manifold approaches. The proposed framework, BT-ROM, utilizes a combination of plain AE and BT self-supervised learning. We use BT self-supervised learning to maximize the information content of the embedding with the latent space through a joint embedding architecture[25]. This model delivers a proficient construction of the latent space; subsequently, it enables us to easily map these latent spaces using regression models. Moreover, this BT-ROM framework can operate on unstructured meshes, which provides flexibility in its application to standard numerical solvers, on-site measurements, experimental data, or a combination of these sources.

The summary of BT-ROM is shown in Supplementary fig. 9. Similar to Supplementary sec. 5.1, the first step is an initialization of $\boldsymbol{\mu}$. In this model, however, $\boldsymbol{\mu}$ is a homogeneous variable not a heterogeneous field. Next, we query a FOM for each parameter $\boldsymbol{\mu}$ in the training set. The third step entails a data compression stage in which we utilize the model developed in Kadeethum et al.[13], and it is summarized in Algorithm 1.

Remark 3 *The procedures derived for BT-ROM - Supplementary sec. 5.2, BBT-ROM - Supplementary sec. 5.3, and in-ROM - Supplementary sec. 5.4 are applicable for both steady-state and transient problems for the sake of compactness. We note that in the steady-state case, the number of a time step is simply one (i.e., $N^t = 1$).*

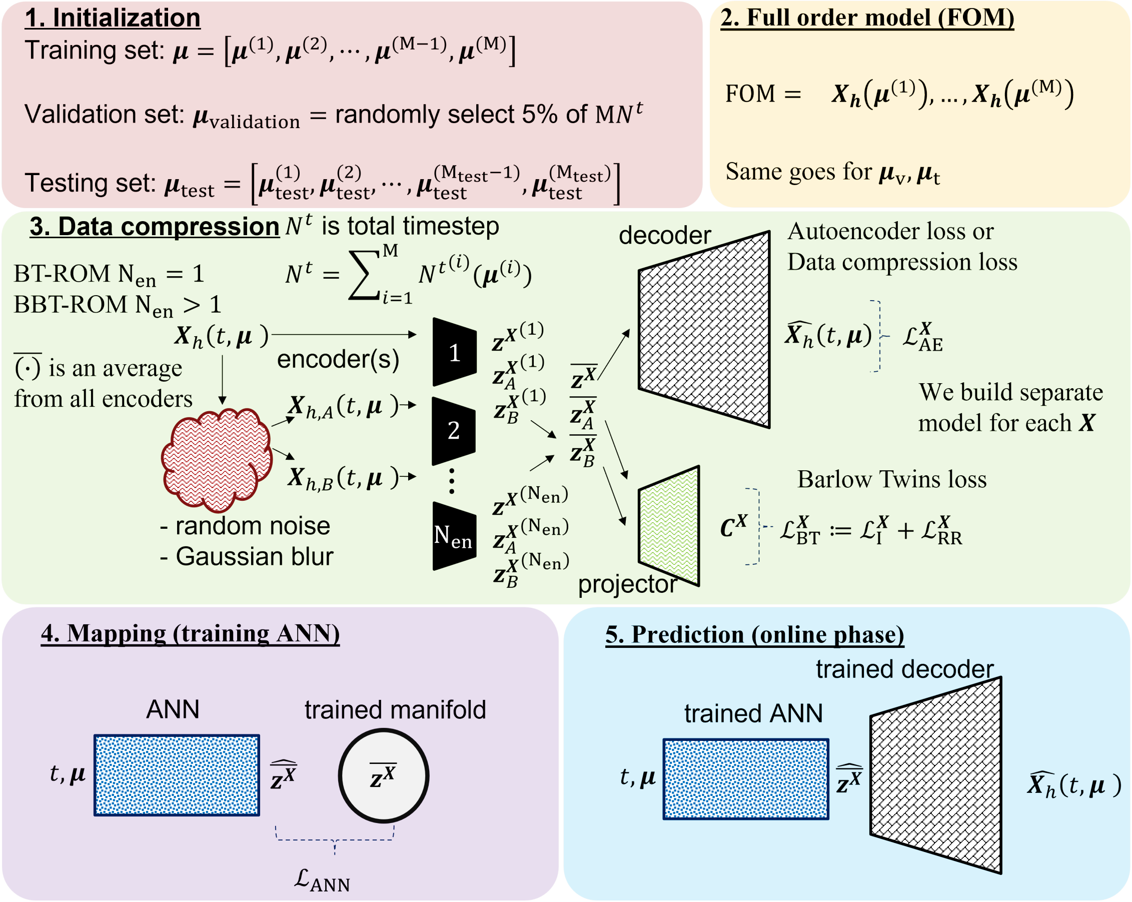

Supplementary fig. 9. Summary of BT- and BBT-ROM: $\boldsymbol{\mu}$ is a set of parameterized spatial parameters (not a field). Our ROM is BT-ROM if the number of encoders $N_{\text{en}} = 1$ while our ROM is BBT if $N_{\text{en}} > 1$.

We reiterate the autoencoder's architecture used for BT-ROM and BBT-ROM in Supplementary tab. 1, and its full description and details can be found in Kadeethum et al.[13]. In short, each linear layer is followed by the LeakyReLU activation

function with a negative slope of 0.2. There are no batch normalization or dropout layers used in this framework.

Supplementary tab. 1. Autoencoder (for both BT-ROM and BBT-ROM) used in this study - corresponding to the third step in Supplementary fig. 9. (input and output sizes are represented by [B, DOF]. B is a batch size, and z is nonlinear manifolds.)

**block**	**input size**	**output size**
1st linear layer	[B, DOF]	[B, int(DOF/2)]
2nd linear layer	[B, int(DOF/2)]	[B, int(DOF/4)]
3rd linear layer	[B, int(DOF/4)]	[B, int(DOF/8)]
4th linear layer	[B, int(DOF/8)]	[B, int(DOF/16)]
5th linear layer	[B, int(DOF/16)]	[B, int(DOF/32)]
1st **bottleneck**	reshape([B, int(DOF/32)])	[B, z]
2nd **bottleneck**	[B, z]	reshape([B, int(DOF/32)])
6th linear layer	[B, int(DOF/32)]	[B, int(DOF/16)]
7th linear layer	[B, int(DOF/16)]	[B, int(DOF/8)]
8th linear layer	[B, int(DOF/8)]	[B, int(DOF/4)]
9th linear layer	[B, int(DOF/4)]	[B, int(DOF/2)]
10th linear layer	[B, int(DOF/2)]	[B, DOF]

The BT-ROM model is composed of one encoder, one decoder, and one projector with their own sets of weight matrices ($\mathbf{W}$) and biases ($\mathbf{b}$). We will discuss BBT-ROM later. The details of the training procedures are provided in Kadeethum et al.[13]. Here, we will give a short summary of the training phase. The training entails two sub-tasks; the first is the training of BT (encoder and projector) - the outer loop with $\mathbf{B}_{\text{outer}}$. The second sub-task is the training of AE (encoder and decoder) - the inner loop with $\mathbf{B}_{\text{inner}}$.

The training begins with distorting our training set by creating $\boldsymbol{X}_{h,A}(t,\boldsymbol{\mu})$ and $\boldsymbol{X}_{h,B}(t,\boldsymbol{\mu})$ from $\boldsymbol{X}(t,\boldsymbol{\mu})$) through the addition of random noise

$$\widetilde{\boldsymbol{X}_{h,A}}(t,\boldsymbol{\mu}),\widetilde{\boldsymbol{X}_{h,B}}(t,\boldsymbol{\mu}) = \boldsymbol{X}(t,\boldsymbol{\mu}) + \varepsilon\,\mathrm{SD}(\boldsymbol{X}(t,\boldsymbol{\mu}))\,\mathscr{G}(0,1) \qquad \text{(Supplementary eq. 24)}$$

where $\widetilde{\boldsymbol{X}_{h,A}}(t,\boldsymbol{\mu}),\widetilde{\boldsymbol{X}_{h,B}}(t,\boldsymbol{\mu})$ are distorted input data resulting from Supplementary eq. 24. The constant ε, which is set to 0.1, determines the noise level, and $\mathscr{G}(0,1)$ is a random value which is sampled from the standard normal distribution with mean and standard deviation of zero and one, respectively. Subsequently, we pass $\widetilde{\boldsymbol{X}_{h,A}}(t,\boldsymbol{\mu}),\widetilde{\boldsymbol{X}_{h,B}}(t,\boldsymbol{\mu})$ through Gaussian blur operation

$$\boldsymbol{X}_{h,A}(t,\boldsymbol{\mu}),\boldsymbol{X}_{h,B}(t,\boldsymbol{\mu}) = \frac{1}{\sqrt{2\pi\,\mathrm{SD}\left(\widetilde{\boldsymbol{X}_{h,A}}(t,\boldsymbol{\mu}),\widetilde{\boldsymbol{X}_{h,B}}(t,\boldsymbol{\mu})\right)^2}}\exp\left(-\frac{\widetilde{\boldsymbol{X}_{h,A}}(t,\boldsymbol{\mu}),\widetilde{\boldsymbol{X}_{h,B}}(t,\boldsymbol{\mu})^2}{2\,\mathrm{SD}\left(\widetilde{\boldsymbol{X}_{h,A}}(t,\boldsymbol{\mu}),\widetilde{\boldsymbol{X}_{h,B}}(t,\boldsymbol{\mu})\right)^2}\right) \qquad \text{(Supplementary eq. 25)}$$

to obtain $\boldsymbol{X}_{h,A}(t,\boldsymbol{\mu})$ and $\boldsymbol{X}_{h,B}(t,\boldsymbol{\mu})$.

For the outer loop, the training BT begins with passing $\boldsymbol{X}_{h,A}(t,\boldsymbol{\mu})$ and $\boldsymbol{X}_{h,B}(t,\boldsymbol{\mu})$ to the encoder (it is noted we have only one encoder) resulting in $\boldsymbol{z}_A^{\boldsymbol{X}}(t,\boldsymbol{\mu})$ and $\boldsymbol{z}_B^{\boldsymbol{X}}(t,\boldsymbol{\mu})$. We then use $\boldsymbol{z}_A^{\boldsymbol{X}}(t,\boldsymbol{\mu})$ and $\boldsymbol{z}_B^{\boldsymbol{X}}(t,\boldsymbol{\mu})$ as an input to the projector resulting in the cross-correlation matrix $\mathbf{C}^{\boldsymbol{X}}(t,\boldsymbol{\mu})$. $\mathbf{C}^{\boldsymbol{X}}(t,\boldsymbol{\mu})$ is a square matrix with the dimensionality of the projector's output. The Barlow Twins loss $\mathscr{L}_{\text{BT}}^{\boldsymbol{X}}$, BT loss, is then calculated using

$$\mathscr{L}_{\text{BT}}^{\boldsymbol{X}} := \mathscr{L}_{\text{I}}^{\boldsymbol{X}} + \mathscr{L}_{\text{RR}}^{\boldsymbol{X}} \qquad \text{(Supplementary eq. 26)}$$

where

$$\mathscr{L}_{\text{I}}^{\boldsymbol{X}} := \sum_i \left(1 - \mathbf{C}_{ii}^{\boldsymbol{X}}(t,\boldsymbol{\mu})\right)^2, \qquad \text{(Supplementary eq. 27)}$$

Algorithm 1 Training autoencoder (AE) with Barlow Twins (BT) self-supervised learning (BT-ROM)

\# Integrate BT into AE architecture to regularize AE's latent spaces $\boldsymbol{z}^{\boldsymbol{X}}$
\###
\# Training data $\boldsymbol{X}_h$ and distorted data $\boldsymbol{X}_{h,A}$, $\boldsymbol{X}_{h,B}$ are input of encoder
\# latent spaces $\boldsymbol{z}^{\boldsymbol{X}}$, $\boldsymbol{z}^{\boldsymbol{X}}_A$, and $\boldsymbol{z}^{\boldsymbol{X}}_B$ are output encoder
\###
\# latent space $\boldsymbol{z}^{\boldsymbol{X}}$ is output of decoder
\# Approximation of $\boldsymbol{X}_h$, i.e., $\widehat{\boldsymbol{X}_h}$ is output of decoder
\###
\# latent spaces $\boldsymbol{z}^{\boldsymbol{X}}_A$ and $\boldsymbol{z}^{\boldsymbol{X}}_B$ are input of projector
\# cross-correlation matrix $\mathbf{C}^{\boldsymbol{X}}$ is output of projector
\###

1: Initialize (or load pre-trained models) encoder, decoder, and projector ▷ size of latent space Q has to be specified.
2: Initialize (or load pre-trained optimizers) three optimizers for each of encoder, decoder, and projector
3: Load training set $\boldsymbol{\mu}$ ▷ the total training data is MN^t
4: Randomly select 5% of MN^t for validation set $\boldsymbol{\mu}_{\text{validation}}$ ▷ the total training data becomes 95% of MN^t
5: Add random noise ▷ see Supplementary eq. 24
6: Add Gaussian blur ▷ see Supplementary eq. 25
7: From step 5 and 6, we obtain $\boldsymbol{X}_{h,A}(t,\boldsymbol{\mu})$ and $\boldsymbol{X}_{h,B}(t,\boldsymbol{\mu})$ from $\boldsymbol{X}(t,\boldsymbol{\mu})$
8: **for each** epoch **do**
9:     Outer loop: training BT ▷ Batch size $\mathbf{B}_{\text{outer}}$
10:     **for each** $\mathbf{B}_{\text{outer}}$ **do**
11:         $\boldsymbol{z}^{\boldsymbol{X}}_A(t,\boldsymbol{\mu}) = \text{encoder}\left(\boldsymbol{X}_{h,A}(t,\boldsymbol{\mu})\right)$
12:         $\boldsymbol{z}^{\boldsymbol{X}}_B(t,\boldsymbol{\mu}) = \text{encoder}\left(\boldsymbol{X}_{h,B}(t,\boldsymbol{\mu})\right)$
13:         $\mathbf{C}^{\boldsymbol{X}}(t,\boldsymbol{\mu}) = \text{projector}\left(\boldsymbol{z}^{\boldsymbol{X}}_A(t,\boldsymbol{\mu}), \boldsymbol{z}^{\boldsymbol{X}}_B(t,\boldsymbol{\mu})\right)$
14:         Calculate BT loss $\mathscr{L}^{\boldsymbol{X}}_{\text{BT}}$ ▷ see Supplementary eq. 26
15:         Back-propagation of BT loss w.r.t. each encoder $(\mathbf{W},\mathbf{b})$ and projector $(\mathbf{W},\mathbf{b})$
16:         Update encoder $(\mathbf{W},\mathbf{b})$ and projector $(\mathbf{W},\mathbf{b})$ using BT optimizer
17:         Update learning rate η_c of BT optimizer ▷ see Supplementary eq. 23
18:         Inner loop: training AE ▷ Batch size $\mathbf{B}_{\text{inner}}$
19:         **for each** $\mathbf{B}_{\text{inner}}$ **do**
20:             $\boldsymbol{z}^{\boldsymbol{X}}(t,\boldsymbol{\mu}) = \text{encoder}\left(\boldsymbol{X}_h(t,\boldsymbol{\mu})\right)$
21:             $\widehat{\boldsymbol{X}_h}(t,\boldsymbol{\mu}) = \text{decoder}\left(\boldsymbol{z}^{\boldsymbol{X}}(t,\boldsymbol{\mu})\right)$
22:             Calculate AE loss $\mathscr{L}^{\boldsymbol{X}}_{\text{AE}}$ (data compression loss) ▷ see Supplementary eq. 29
23:             Back-propagation of AE loss w.r.t. each encoder $(\mathbf{W},\mathbf{b})$ and decoder $(\mathbf{W},\mathbf{b})$
24:             Update encoder $(\mathbf{W},\mathbf{b})$ and decoder $(\mathbf{W},\mathbf{b})$ using AE optimizer
25:             Update learning rate η_c of AE optimizer ▷ see Supplementary eq. 23
26:         **end for**
27:     **end for**
28: **end for**

This algorithm only reflects the third step in Supplementary fig. 9.

and

$$\mathscr{L}^{\boldsymbol{X}}_{\text{RR}} := \lambda \sum_i \sum_{j \neq i} \mathbf{C}^{\boldsymbol{X}}_{ij}(t,\boldsymbol{\mu})^2. \qquad \text{(Supplementary eq. 28)}$$

Here, $\mathbf{C}^{\boldsymbol{X}}_{ii}(t,\boldsymbol{\mu})$ denotes the i-th diagonal entry of $\mathbf{C}^{\boldsymbol{X}}(t,\boldsymbol{\mu})$, λ is set to 5×10^{-3}, and $\mathbf{C}^{\boldsymbol{X}}_{ij}$ are off-diagonal entries of $\mathbf{C}^{\boldsymbol{X}}$. We follow the training procedures also used in Supplementary sec. 5.1 to adjust learnable parameters of encoder(W and b) and projector(W and b). The learning rate (η) is calculated by Supplementary eq. 23, $\eta_{\min}$ is set as 1×10^{-16}, and $\eta_{\max}$ is selected as 1×10^{-4}.

For the inner loop, the training of AE starts with obtaining $\boldsymbol{z}^{\boldsymbol{X}}(t,\boldsymbol{\mu})$ by passing $\boldsymbol{X}_h(t,\boldsymbol{\mu})$ to the encoder. We then use $\boldsymbol{z}^{\boldsymbol{X}}(t,\boldsymbol{\mu})$ to reconstruct $\widehat{\boldsymbol{X}}_h(t,\boldsymbol{\mu})$ through the decoder. Subsequently, we calculate our data compression loss or AE loss ($\mathscr{L}^{\boldsymbol{X}}_{\text{AE}}$) using

$$\mathscr{L}_{\mathrm{AE}}^{\boldsymbol{X}} := \mathrm{MSE}^{\boldsymbol{X}} = \frac{1}{\mathrm{M}N^t}\sum_{i=1}^{\mathrm{M}}\sum_{k=0}^{N^t}\left|\widehat{\boldsymbol{X}}_h\left(t^k,\boldsymbol{\mu}^{(i)}\right) - \boldsymbol{X}_h\left(t^k,\boldsymbol{\mu}^{(i)}\right)\right|^2. \quad \text{(Supplementary eq. 29)}$$

Again, we use ADAM to adjust learnable parameters of the encoder(W and b) and decoder(W and b) according to the gradient of Supplementary eq. 29. The η_c is adjusted by Supplementary eq. 23, and we use $\eta_{\min} = 1\times10^{-16}$, and $\eta_{\max} = 1\times10^{-5}$. Again, to prevent our networks from overfitting behavior, we follow early stopping and generalized cross-validation criteria[37,38].

For step 4th in Supplementary fig. 9, we train artificial neural networks (ANN) to map between $(t,\boldsymbol{\mu})$ and $\boldsymbol{z}^{\boldsymbol{X}}(t,\boldsymbol{\mu})$, the established manifolds obtained during the third step shown in Supplementary fig. 9. Following Kadeethum et al.[12,13,43], our ANN has five hidden layers, and each layer has seven neurons. We use tanh as our activation function. Here, we use a mean squared error ($\mathrm{MSE}^{z^{\boldsymbol{X}}}$) as the metric of our network loss function, defined as follows

$$\mathscr{L}_{\mathrm{ANN}}^{\boldsymbol{X}} := \mathrm{MSE}^{z^{\boldsymbol{X}}} = \frac{1}{\mathrm{M}N^t}\sum_{i=1}^{\mathrm{M}}\sum_{k=0}^{N^t}\left|\widehat{z}^{\boldsymbol{X}}\left(t^k,\boldsymbol{\mu}^{(i)}\right) - z^{\boldsymbol{X}}\left(t^k,\boldsymbol{\mu}^{(i)}\right)\right|^2. \quad \text{(Supplementary eq. 30)}$$

To minimize Supplementary eq. 30, i.e., train our ANN, we use the ADAM algorithm to adjust each neuron W and b, set a batch size of 32, a learning rate of 0.001, a number of epoch of 10,000, and normalize both our input $(t,\boldsymbol{\mu})$ and output $(z^{\boldsymbol{X}})$ to $[0,1]$. For all the training, BT and AE, to prevent our networks from overfitting behavior, we follow early stopping and generalized cross-validation criteria[37,44], i.e., instead of literally stopping our training cycle, we only save the set of trained weight and bias to be used in the online phase when the current validation loss is lower than the lowest validation from all the previous training cycle.

During the online phase - see Supplementary fig. 9, we utilize the trained ANN and the trained decoder to approximate $\widehat{\boldsymbol{X}}_h(\cdot;t,\boldsymbol{\mu})$ for each inquiry (i.e., a pair of $(t,\boldsymbol{\mu})$) through

$$\widehat{z}^{\boldsymbol{X}}(\cdot;t,\boldsymbol{\mu}) = \mathrm{ANN}(t,\boldsymbol{\mu}), \quad \text{(Supplementary eq. 31)}$$

and, subsequently,

$$\widehat{\boldsymbol{X}}_h(\cdot;t,\boldsymbol{\mu}) = \mathrm{decoder}\left(\widehat{z}^{\boldsymbol{X}}(\cdot;t,\boldsymbol{\mu})\right). \quad \text{(Supplementary eq. 32)}$$

Supplementary sec. 5.3 Reduced order modeling with boosting Barlow Twins self-supervised learning

We now derive an enhanced model of BT-ROM through boosting technique[45,46]. We adapt the boosting procedures proposed in Krishna et al.[47] to our BT-ROM, which is named BBT-ROM hereafter. We propose this model because the primary challenge for applying machine learning techniques to the physics-based problems with point source (or Dirac delta distribution); for instance, contact problems or subsurface flow with wells, is how to deal with imbalanced training data[48,49].

The summary of the BBT-ROM is presented in Supplementary fig. 9. We note that the only difference from the BT-ROM model lies within the third step or the data compression step. To elaborate, the model is BT-ROM when we have only one encoder ($\mathrm{N_{en}} = 1$), and the model is BBT-ROM when we have more than one encoder ($\mathrm{N_{en}} > 1$). Both BT-ROM and BBT-ROM have only one decoder and one projector. The training procedures of the BBT-ROM are provided in Algorithm 2. For the BBT-ROM, we do not train our data compression framework using the whole training set, but only a sub-sample of it (i.e., $x_{\mathrm{sub}}\%$ of training set). We sub-sample our training set based on the weighted vector $\boldsymbol{w}_{\mathrm{sample}}$ defined as

$$\boldsymbol{w}_{\mathrm{sample}} = \begin{cases} \frac{\mathbf{1}}{\mathrm{M}N^t}, & \text{for the first encoder} \\ \frac{\left|\widehat{\boldsymbol{X}}_h\left(t^k,\boldsymbol{\mu}^{(i)}\right) - \boldsymbol{X}_h\left(t^k,\boldsymbol{\mu}^{(i)}\right)\right|^2}{\sum_{i=1}^{\mathrm{M}}\sum_{k=0}^{N^t}\left|\widehat{\boldsymbol{X}}_h\left(t^k,\boldsymbol{\mu}^{(i)}\right) - \boldsymbol{X}_h\left(t^k,\boldsymbol{\mu}^{(i)}\right)\right|^2}, & \text{otherwise,} \end{cases} \quad \text{(Supplementary eq. 33)}$$

where $\mathbf{1}$ is a unit vector of length $\mathrm{M}N^t$. We note that all training samples are weighted equally for the first encoder. For the subsequent encoder(s), the weighted vector is updated according to an $\mathrm{encoder}_{i-1}$'s performance and used for the training of $\mathrm{encoder}_i$. With this procedure, an $\mathrm{encoder}_i$ is forced to learn training samples in which $\mathrm{encoder}_{i-1},\ldots,\mathrm{encoder}_1$ fail to excel.

Given that we have $\mathrm{N_{en}}$ encoders and $\mathrm{N_{en}} > 1$, but we have only one decoder and one projector, we take an arithmetic average over the latent manifolds produced by each encoder as

$$\bar{z}^{\boldsymbol{X}}_h(t,\boldsymbol{\mu}) = \frac{\sum_{j=1}^{\mathrm{N_{en}}} z^{\boldsymbol{X}}_j(t,\boldsymbol{\mu})}{\mathrm{N_{en}}}, \quad \bar{z}^{\boldsymbol{X}}_{h,A}(t,\boldsymbol{\mu}) = \frac{\sum_{j=1}^{\mathrm{N_{en}}} z^{\boldsymbol{X}}_{j,h,A}(t,\boldsymbol{\mu})}{\mathrm{N_{en}}}, \quad \bar{z}^{\boldsymbol{X}}_{h,B}(t,\boldsymbol{\mu}) = \frac{\sum_{j=1}^{\mathrm{N_{en}}} z^{\boldsymbol{X}}_{j,h,B}(t,\boldsymbol{\mu})}{\mathrm{N_{en}}}. \quad \text{(Supplementary eq. 34)}$$

We note that, as also stated by Krishna et al.[47], other types of average or ensemble models could be also used instead of an arithmetic average. However, we will leave those investigations for our future works. Following with the training of BBT-ROM or data compression part, the framework follows similar procedures illustrated in Supplementary fig. 9.

Supplementary sec. 5.4 Intrusive reduced order modeling through Galerkin projection

We here briefly discuss the intrusive reduced order modeling through Galerkin projection (in-ROM). Comprehensive detailed, as well as implementations, could be found in Kadeethum et al.[43]. Again, The procedures derived here are applicable for both steady-state and transient problems. For the steady-state case, $N^t = 1$. In contrast to the models described in Supplementary sec. 5.1, Supplementary sec. 5.2, and Supplementary sec. 5.3, the in-ROM relies on linear manifolds. The linear manifolds are derived through proper orthogonal decomposition (POD)[43,50,51]. This framework utilizes the same procedures, steps 1 and 2, outlined in Supplementary fig. 9. In the third step, we perform a POD to compress the training data produced from the second step. The POD method begins with collecting all snapshots in a matrix

$$\mathbb{S}_{\boldsymbol{X}} = \left[\mathbb{S}^{(1)}_{\boldsymbol{X}}, \cdots, \mathbb{S}^{(\mathrm{M})}_{\boldsymbol{X}}\right] \in \mathbb{R}^{N_h^{\boldsymbol{X}} \times N^t \mathrm{M}}, \quad \text{(Supplementary eq. 35)}$$

by horizontally stacking all matrices $\mathbb{S}^{(i)}_{\boldsymbol{X}}$, $i = 1, \dots, \mathrm{M}$. We then perform the singular value decomposition (SVD) of $\mathbb{S}_{\boldsymbol{X}}$ as

$$\mathbb{S}_{\boldsymbol{X}} = \mathbb{W} \begin{bmatrix} \mathbb{D} & 0 \\ 0 & 0 \end{bmatrix} \mathbb{Z}^\top \quad \text{(Supplementary eq. 36)}$$

where $\mathbb{W} = \left[\mathbf{w}_1, \cdots, \mathbf{w}_{N_h^{\boldsymbol{X}}}\right] \in \mathbb{R}^{N_h^{\boldsymbol{X}} \times N_h^{\boldsymbol{X}}}$ and $\mathbb{Z} = [\mathbf{z}_1, \cdots, \mathbf{z}_{N^t\mathrm{M}}] \in \mathbb{R}^{N^t\mathrm{M} \times N^t\mathrm{M}}$ are orthogonal matrices, $\mathbb{D} = \mathrm{diag}(\sigma_1, \cdots, \sigma_r) \in \mathbb{R}^{r \times r}$ is a diagonal matrix, with singular values $\sigma_1 \geq \sigma_2 \geq \cdots \geq \sigma_r > 0$. Here, r is the number of non-zero singular values and $r \leq \min\left\{N_h^{\boldsymbol{X}}, N^t\mathrm{M}\right\}$. The columns of $\mathbb{W}$ are called left singular vectors of $\mathbb{S}$, and the columns of $\mathbb{Z}$ are called right singular vectors of $\mathbb{S}$. To carry out a dimensionality reduction, the POD basis of rank $\mathrm{N} \ll r$ consists of the first N left singular vectors of $\mathbb{S}$, and it has the property of minimizing the projection error defined by

$$\{\mathbf{w}_1, \cdots, \mathbf{w}_{\mathrm{N}}\} = \arg\min \left\{ \varepsilon(\tilde{\mathbf{w}}_1, \cdots, \tilde{\mathbf{w}}_{\mathrm{N}}) = \sum_{i=1}^{\mathrm{M}} \sum_{k=0}^{N^t} \left\| \boldsymbol{X}_h\left(\cdot; t^k, \boldsymbol{\mu}^{(i)}\right) - \sum_{n=1}^{\mathrm{N}} \left(\boldsymbol{X}_h\left(\cdot; t^k, \boldsymbol{\mu}^{(i)}\right), \tilde{\mathbf{w}}_n\right)_u \tilde{\mathbf{w}}_n \right\|_u^2 \right\}$$

(Supplementary eq. 37)

among all the orthonormal bases $\{\tilde{\mathbf{w}}_1, \cdots, \tilde{\mathbf{w}}_{\mathrm{N}}\} \subset \mathbb{R}^{N_h^{\boldsymbol{X}}}$. Here $(\cdot,\cdot)_{\boldsymbol{X}}$ denotes an inner product for the space of the primary variable $\boldsymbol{X}$, while $\|\cdot\|_{\boldsymbol{X}}$ its induced norm. The reduced basis space $\mathscr{U}_{\mathrm{N}}$ is then defined as the span of $\{\mathbf{w}_1, \cdots, \mathbf{w}_{\mathrm{N}}\}$, and $\{q_1, \cdots, q_{\mathrm{N}}\}$ denote the basis functions spanning $\mathscr{P}_{\mathrm{N}}$.

During the online phase, one carries out a time stepping as follows: given $\widehat{\theta}^{\boldsymbol{X}}_k(t^0, \boldsymbol{\mu})$ for every time step t^n find reduced coefficients $\widehat{\theta}^{\boldsymbol{X}}_k(t^n, \boldsymbol{\mu})$ such that the reconstructed solutions

$$\widehat{\boldsymbol{X}}^n_h := \widehat{\boldsymbol{X}}_h(\cdot; t^n, \boldsymbol{\mu}) = \sum_{k=1}^{\mathrm{N}} \widehat{\theta}^{\boldsymbol{X}}_k(t^n, \boldsymbol{\mu}) \mathbf{w}_k,$$

are solution to the following Galerkin method

$$\left\{ \mathscr{A}_{\boldsymbol{X}}\left((\widehat{\boldsymbol{X}}^n_h, \widehat{p}^n_h), \mathbf{w}_k\right) = \mathscr{L}_{\boldsymbol{X}}(\mathbf{w}_k), \quad \forall k = 1, \dots, N, \right.$$

Supplementary sec. 5.5 Residual vs. iterations analysis

We present a residual as a function of the number of iterations for Example 2.2: Indentation radius and indentation depth are parameters in Supplementary Figure 10. We observe that results with the ROM-assisted results have a lower starting point of the residual than the default initialization (i.e., the starting point of the ROM-assisted model is approximately the third iteration for default initialization). However, the slope comparing between the starting point of ROM-assisted until the convergence point and the third iteration of the default initialization until the convergence point is approximately similar. Hence, we hypothesize that ROM-assisted models help the nonlinear solver by giving a smaller initial residual, not accelerating a rate.

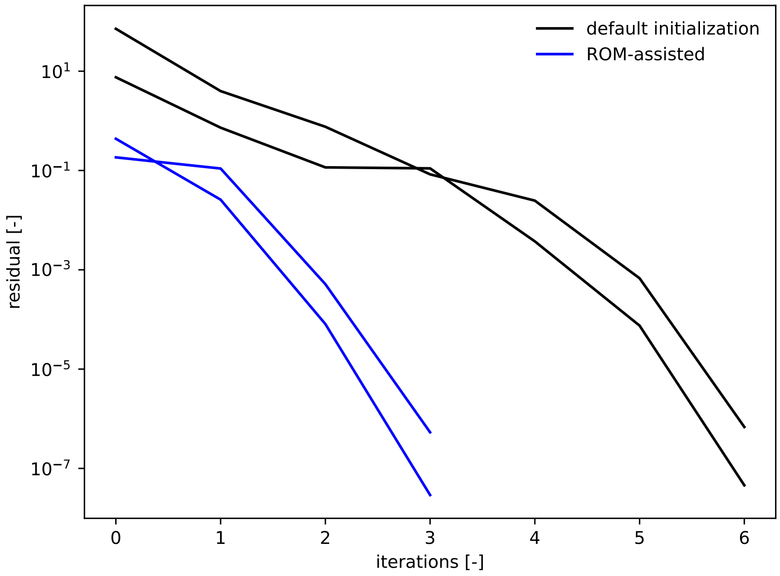

Supplementary fig. 10. Residual as a function of iterations plot for Example 2.2: Indentation radius and indentation depth are parameters

References

1. Farthing, M. & Ogden, F. Numerical solution of richards' equation: A review of advances and challenges. Soil Sci. Soc. Am. J. **81**, 1257–1269 (2017).
2. Kumar, N. & DasGupta, A. On the contact problem of an inflated spherical hyperelastic membrane. Int. J. Non-Linear Mech. **57**, 130–139 (2013).
3. Luo, Y., Li, M. & Kang, Z. Topology optimization of hyperelastic structures with frictionless contact supports. Int. J. Solids Struct. **81**, 373–382 (2016).
4. Nezamabadi, S., Zahrouni, H. & Yvonnet, J. Solving hyperelastic material problems by asymptotic numerical method. Comput. mechanics **47**, 77–92 (2011).
5. Jeremy, B. Numerical Tours of Computational Mechanics with FEniCS, DOI: 10.5281/zenodo.1287832 (2018).
6. Balay, S. et al. PETSc Users Manual. Tech. Rep. ANL-95/11 - Revision 3.10, Argonne National Laboratory (2018).
7. Jackson, M. et al. Reservoir modeling for flow simulation by use of surfaces, adaptive unstructured meshes, and an overlapping-control-volume finite-element method. SPE Reserv. Eval. & Eng. **18**, 115–132 (2015).
8. Gomes, J. et al. A force-balanced control volume finite element method for multi-phase porous media flow modelling. Int. J. for Numer. Methods Fluids **83**, 431–445 (2017).
9. Salinas, P. et al. Improving the convergence behaviour of a fixed-point-iteration solver for multiphase flow in porous media. Int. J. for Numer. Methods Fluids **84**, 466–476 (2017).
10. Salinas, P. et al. Improving the robustness of the control volume finite element method with application to multiphase porous media flow. Int. J. for Numer. Methods Fluids **85**, 235–246 (2017).
11. Pavlidis, D. et al. Two-and three-phase horizontal slug flow simulations using an interface-capturing compositional approach. Int. J. Multiph. Flow **67**, 85–91 (2014).
12. Kadeethum, T. et al. Non-intrusive reduced order modeling of natural convection in porous media using convolutional autoencoders: comparison with linear subspace techniques. Adv. Water Resour. 104098 (2022).
13. Kadeethum, T. et al. Reduced order modeling with barlow twins self-supervised learning: Navigating the space between linear and nonlinear solution manifolds. arXiv preprint arXiv:2202.05460 (2022).
14. Brooks, R. H. Hydraulic properties of porous media (Colorado State University, 1965).

15. Silva, V., Salinas, P., Jackson, M. & Pain, C. Machine learning acceleration for nonlinear solvers applied to multiphase porous media flow. Comput. Methods Appl. Mech. Eng. **384**, 113989 (2021).
16. Francfort, G. & Marigo, J.-J. Revisiting brittle fracture as an energy minimization problem. J. Mech. Phys. Solids **46**, 1319–1342 (1998).
17. Bourdin, B., Francfort, G. & Marigo, J.-J. Numerical experiments in revisited brittle fracture. J. Mech. Phys. Solids **48**, 797–826 (2000).
18. Griffith, A. The phenomena of rupture and flow in solids. Philos. Trans. R. Soc. Lond. **221**, 163–198 (1921).
19. Ambrosio, L. & Tortorelli, V. Approximation of functionals depending on jumps by elliptic functionals via γ-convergence. Comm. Pure Appl. Math. **43**, 999–1036 (1990).
20. Heister, T., Wheeler, M. F. & Wick, T. A primal-dual active set method and predictor-corrector mesh adaptivity for computing fracture propagation using a phase-field approach. Comput. Methods Appl. Mech. Engrg. **290**, 466–495 (2015).
21. Lee, S., Wheeler, M. F. & Wick, T. Pressure and fluid-driven fracture propagation in porous media using an adaptive finite element phase field model. Comput. Methods Appl. Mech. Eng. **305**, 111 – 132 (2016).
22. Arndt, D. et al. The `deal.II` library, version 9.1. J. Numer. Math. DOI: 10.1515/jnma-2019-0064 (2019). Accepted.
23. Kadeethum, T. et al. A framework for data-driven solution and parameter estimation of pdes using conditional generative adversarial networks. Nat. Comput. Sci. **1**, 819–829, DOI: https://doi.org/10.1038/s43588-021-00171-3 (2021).
24. Kadeethum, T. et al. Continuous conditional generative adversarial networks for data-driven solutions of poroelasticity with heterogeneous material properties. Comput. Geosci. **167**, 105212 (2022).
25. Zbontar, J., Jing, L., Misra, I., LeCun, Y. & Deny, S. Barlow twins: Self-supervised learning via redundancy reduction. arXiv preprint arXiv:2103.03230 (2021).
26. Shen, Y., Gu, J., Tang, X. & Zhou, B. Interpreting the latent space of gans for semantic face editing. In Proceedings of the IEEE/CVF Conference on Computer Vision and Pattern Recognition, 9243–9252 (2020).
27. Mirza, M. & Osindero, S. Conditional generative adversarial nets. arXiv preprint arXiv:1411.1784 (2014).
28. Chen, X. et al. Infogan: Interpretable representation learning by information maximizing generative adversarial nets. arXiv preprint arXiv:1606.03657 (2016).
29. Isola, P., Zhu, J., Zhou, T. & Efros, A. Image-to-image translation with conditional adversarial networks. In Proceedings of the IEEE conference on computer vision and pattern recognition, 1125–1134 (2017).
30. Ma, L. et al. Pose guided person image generation. arXiv preprint arXiv:1705.09368 (2017).
31. Ronneberger, O., Fischer, P. & Brox, T. U-net: Convolutional networks for biomedical image segmentation. In International Conference on Medical image computing and computer-assisted intervention, 234–241 (Springer, 2015).
32. Demir, U. & Unal, G. Patch-based image inpainting with generative adversarial networks. arXiv preprint arXiv:1803.07422 (2018).
33. Arjovsky, M., Chintala, S. & Bottou, L. Wasserstein generative adversarial networks. In International conference on machine learning, 214–223 (PMLR, 2017).
34. Gulrajani, I., Ahmed, F., Arjovsky, M., Dumoulin, V. & Courville, A. Improved training of wasserstein gans. arXiv preprint arXiv:1704.00028 (2017).
35. Kingma, D. & Ba, J. Adam: A method for stochastic optimization. arXiv preprint arXiv:1412.6980 (2014).
36. Loshchilov, I. & Hutter, F. Sgdr: Stochastic gradient descent with warm restarts. arXiv preprint arXiv:1608.03983 (2016).
37. Prechelt, L. Early stopping-but when? In Neural Networks: Tricks of the trade, 55–69 (Springer, 1998).
38. Prechelt, L. Automatic early stopping using cross validation: quantifying the criteria. Neural Networks **11**, 761–767 (1998).
39. Fresca, S., Dede, L. & Manzoni, A. A comprehensive deep learning-based approach to reduced order modeling of nonlinear time-dependent parametrized pdes. J. Sci. Comput. **87**, 1–36 (2021).
40. Kim, Y., Choi, Y., Widemann, D. & Zohdi, T. A fast and accurate physics-informed neural network reduced order model with shallow masked autoencoder. J. Comput. Phys. 110841 (2021).
41. Willcox, K. & Peraire, J. Balanced model reduction via the proper orthogonal decomposition. AIAA journal **40**, 2323–2330 (2002).

42. Choi, Y., Coombs, D. & Anderson, R. Sns: a solution-based nonlinear subspace method for time-dependent model order reduction. SIAM J. on Sci. Comput. **42**, A1116–A1146 (2020).

43. Kadeethum, T., Ballarin, F. & Bouklas, N. Data-driven reduced order modeling of poroelasticity of heterogeneous media based on a discontinuous galerkin approximation. GEM-International J. on Geomathematics **12**, 1–45 (2021).

44. Hesthaven, J. & Ubbiali, S. Non-intrusive reduced order modeling of nonlinear problems using neural networks. J. Comput. Phys. **363**, 55–78 (2018).

45. Schapire, R. The boosting approach to machine learning: An overview. Nonlinear estimation classification 149–171 (2003).

46. Schapire, R. & Freund, Y. Boosting: Foundations and algorithms. Kybernetes (2013).

47. Krishna, S., Tholeti, T. & Kalyani, S. How to boost autoencoders? arXiv preprint arXiv:2110.15307 (2021).

48. Zong, W., Huang, G. & Chen, Y. Weighted extreme learning machine for imbalance learning. Neurocomputing **101**, 229–242 (2013).

49. Thabtah, F., Hammoud, S., Kamalov, F. & Gonsalves, A. Data imbalance in classification: Experimental evaluation. Inf. Sci. **513**, 429–441 (2020).

50. Lumley, J. The structure of inhomogeneous turbulent flows. Atmospheric turbulence radio wave propagation (1967).

51. Hesthaven, J., Rozza, G., Stamm, B. et al. Certified reduced basis methods for parametrized partial differential equations (Springer, 2016).

Algorithm 2 Training boosting autoencoder (AE) with Barlow Twins (BT) self-supervised learning (BBT-ROM)

Integrate boosting into the BT-ROM, i.e, BBT-ROM
###
Training data $\boldsymbol{X}_h$ and distorted data $\boldsymbol{X}_{h,A}$, $\boldsymbol{X}_{h,B}$ are input of encoder
latent spaces $\boldsymbol{z}^{\boldsymbol{X}}$, $\boldsymbol{z}^{\boldsymbol{X}}_A$, and $\boldsymbol{z}^{\boldsymbol{X}}_B$ are output encoder
###
latent space $\boldsymbol{z}^{\boldsymbol{X}}$ is output of decoder
Approximation of $\boldsymbol{X}_h$, i.e., $\widehat{\boldsymbol{X}_h}$ is output of decoder
###
latent spaces $\boldsymbol{z}^{\boldsymbol{X}}_A$ and $\boldsymbol{z}^{\boldsymbol{X}}_B$ are input of projector
cross-correlation matrix $\mathbf{C}^{\boldsymbol{X}}$ is output of projector
###

1: Specify number of encoders $\mathrm{N_{en}}$ ▷ We have $\mathrm{N_{en}}$ encoders, but one decoder, and one projector.
2: Initialize (or load pre-trained models) encoders, decoder, and projector ▷ size of latent space Q has to be specified.
3: Initialize (or load pre-trained optimizers) $2 \times \mathrm{N_{en}}$ optimizers, two for each encoder, one optimizer for decoder, and one optimizer for projector ▷ Each encoder has two optimizers for BT and AE training.
4: Load training set $\boldsymbol{\mu}$ ▷ the total training data is $\mathrm{M}N^t$
5: Randomly select 5% of $\mathrm{M}N^t$ for validation set $\boldsymbol{\mu}_{\text{validation}}$ ▷ the total training data becomes 95% of $\mathrm{M}N^t$
6: Add random noise ▷ see Supplementary eq. 24
7: Add Gaussian blur ▷ see Supplementary eq. 25
8: From step 6 and 7, we obtain $\boldsymbol{X}_{h,A}(t,\boldsymbol{\mu})$ and $\boldsymbol{X}_{h,B}(t,\boldsymbol{\mu})$ from $\boldsymbol{X}_h(t,\boldsymbol{\mu})$
9: **for** $i \in [1,\ldots,\mathrm{N_{en}}]$ **do** ▷ We train an encoder$_i$ sequentially
10: Update (or initialize) the weighted vector ($\boldsymbol{w}_{\text{sample}}$) for sub-sample training set ▷ see Supplementary eq. 33
11: **for each** epoch **do**
12: Sub-sample x_{sub}% of training set according to $\boldsymbol{w}_{\text{sample}}$
13: <u>Outer loop: training BT</u> ▷ Batch size $\mathbf{B}_{\text{outer}}$
14: **for each** $\mathrm{B} \in \mathbf{B}_{\text{outer}}$ **do**
15: **for each** encoder **do** ▷ We only calculate gradient of an encoder$_i$ corresponding to line 9
16: $\boldsymbol{z}^{\boldsymbol{X}}_{j,A}(t,\boldsymbol{\mu}) = \text{encoder}_j\left(\boldsymbol{X}_{h,A}(t,\boldsymbol{\mu})\right)$
17: $\boldsymbol{z}^{\boldsymbol{X}}_{j,B}(t,\boldsymbol{\mu}) = \text{encoder}_j\left(\boldsymbol{X}_{h,B}(t,\boldsymbol{\mu})\right)$
18: **end for**
19: Calculate $\bar{\boldsymbol{z}}^{\boldsymbol{X}}_A(t,\boldsymbol{\mu})$ and $\bar{\boldsymbol{z}}^{\boldsymbol{X}}_B(t,\boldsymbol{\mu})$ ▷ see Supplementary eq. 34
20: $\mathbf{C}^{\boldsymbol{X}}(t,\boldsymbol{\mu}) = \text{projector}\left(\bar{\boldsymbol{z}}^{\boldsymbol{X}}_A(t,\boldsymbol{\mu}), \bar{\boldsymbol{z}}^{\boldsymbol{X}}_B(t,\boldsymbol{\mu})\right)$
21: Calculate BT loss $\mathscr{L}^{\boldsymbol{X}}_{\text{BT}}$ ▷ see Supplementary eq. 26
22: Back-propagation of BT loss w.r.t. encoder$_i$ $(\mathbf{W},\mathbf{b})$ and projector $(\mathbf{W},\mathbf{b})$
23: Update encoder$_i$ $(\mathbf{W},\mathbf{b})$ using encoder$_i$ optimizer and projector $(\mathbf{W},\mathbf{b})$ using projector optimizer
24: Update learning rate η_c of encoder$_i$ optimizer and projector optimizer ▷ see Supplementary eq. 23
25: <u>Inner loop: training AE</u> ▷ Batch size $\mathbf{B}_{\text{inner}}$
26: **for each** $\mathrm{B} \in \mathbf{B}_{\text{inner}}$ **do**
27: **for each** encoder **do** ▷ We only calculate gradient of an encoder$_i$ corresponding to line 27
28: $\boldsymbol{z}^{\boldsymbol{X}}_j(t,\boldsymbol{\mu}) = \text{encoder}_j(\boldsymbol{X}_h(t,\boldsymbol{\mu}))$
29: **end for**
30: Calculate $\bar{\boldsymbol{z}}^{\boldsymbol{X}}(t,\boldsymbol{\mu})$ ▷ see Supplementary eq. 34
31: $\widehat{\boldsymbol{X}_h}(t,\boldsymbol{\mu}) = \text{decoder}\left(\bar{\boldsymbol{z}}^{\boldsymbol{X}}(t,\boldsymbol{\mu})\right)$
32: Calculate AE loss $\mathscr{L}^{\boldsymbol{X}}_{\text{AE}}$ (data compression loss) ▷ see Supplementary eq. 29
33: Back-propagation of AE loss w.r.t. encoder$_i$ $(\mathbf{W},\mathbf{b})$ and decoder $(\mathbf{W},\mathbf{b})$
34: Update encoder$_i$ $(\mathbf{W},\mathbf{b})$ using encoder$_i$ optimizer and decoder $(\mathbf{W},\mathbf{b})$ using decoder optimizer
35: Update learning rate η_c of encoder$_i$ optimizer and decoder optimizer ▷ see Supplementary eq. 23
36: **end for**
37: **end for**
38: **end for**
39: **end for**

This algorithm only reflects the third step in Supplementary fig. 9.
